# Sensitivity of standardised radiomics algorithms to mask generation across different software platforms

**DOI:** 10.1038/s41598-023-41475-w

**Published:** 2023-09-02

**Authors:** Philip Whybra, Emiliano Spezi

**Affiliations:** School of Engineering, Cardiff University, Cardiff, CF24 3AA UK

**Keywords:** Biomedical engineering, Image processing

## Abstract

The field of radiomics continues to converge on a standardised approach to image processing and feature extraction. Conventional radiomics requires a segmentation. Certain features can be sensitive to small contour variations. The industry standard for medical image communication stores contours as coordinate points that must be converted to a binary mask before image processing can take place. This study investigates the impact that the process of converting contours to mask can have on radiomic features calculation. To this end we used a popular open dataset for radiomics standardisation and we compared the impact of masks generated by importing the dataset into 4 medical imaging software. We interfaced our previously standardised radiomics platform with these software using their published application programming interface to access image volume, masks and other data needed to calculate features. Additionally, we used super-sampling strategies to systematically evaluate the impact of contour data pre processing methods on radiomic features calculation. Finally, we evaluated the effect that using different mask generation approaches could have on patient clustering in a multi-center radiomics study. The study shows that even when working on the same dataset, mask and feature discrepancy occurs depending on the contour to mask conversion technique implemented in various medical imaging software. We show that this also affects patient clustering and potentially radiomic-based modelling in multi-centre studies where a mix of mask generation software is used. We provide recommendations to negate this issue and facilitate reproducible and reliable radiomics.

## Introduction

Traditional radiomic methods seek imaging biomarkers that quantify disease through analysis of regions of interest (ROIs) in medical imaging. With radiomics, models built from relevant and explainable imaging biomarkers could provide additional insights to guide clinical decisions and facilitate personalised treatment strategies^1–4^. Radiomics is a growing topic of interest in oncology, which extensively uses imaging throughout diagnosis and treatment. In oncology, radiomics features usually come from ROIs defining tumours or organs at risk.

Since the term radiomics entered the literature lexicon, numerous studies have explored—and addressed—many challenges surrounding the reproducibility of key features that describe shape, statistics, and texture^5,6^. With the traditional radiomics approach, assessing and selecting *reproducible* features as potential imaging biomarkers is essential for model generalisability. Only models built with reproducible features can survive external validation and see routine use in the clinic.

To enable reproducibility of radiomic studies, the Image Biomarker Standardisation Initiative (IBSI)^7^ represents a significant effort to produce a set of standardised reference values for a number of feature families. The IBSI determined benchmarks in two phases, iterating towards consensus-based reference values considering a variety of extraction settings. These benchmarks were developed using a digital phantom and a single lung computed tomography (CT) image with a gross tumour volume (GTV) segmentation. An extensive reference manual was produced detailing the image processing steps for extraction. Finally, a cohort of 51 patients was used in a third validation phase to evaluate the reproducibility of software from different participants, after the benchmarking phases were completed. The validation phase showed excellent reproducibility for successfully benchmarked features.

A number of studies have investigated the effect of segmentation on radiomic features. Typically studies test the individual robustness of features holding extraction settings constant, whilst changing the contour defining the ROI. Often, this is tested in the context of manual vs. automatic methods. For instance Belli et al.^8^ quantified the robustness of radiomic features on pancreatic cancer delineated on 18F-fluorodeoxyglucose (FDG) positron emission tomography (PET) were FDG-based contours were delineated following manual, semi-automatic and automatic segmentation methods. Haarburger et al.^9^ analysed the reproducibility of radiomic features using expert manual segmentations and neural network-based CT segmentations on lung, kidney and liver lesions. Fiset et al.^10^ evaluated the stability of radiomic features from T2-weighted MRI of cervical cancer using test-retest, simulated MRI data and inter-observer segmentation. Due to intra- and inter- variability of manual segmentation, semi- and automated methods have been shown to facilitate more repeatable radiomic results^6^. Notably, results from a phantom study in PET by Pfaehler et al.^11^ found that segmentation of smaller volumes led to lower repeatability of radiomic feature values. In essence, the effect of segmentation on features is emphasised in volumes with fewer voxels.

In addition to comparing manual vs automatic segmentation, differences in delineation can be simulated by computationally manipulating the ROI binary mask through adaptation of the volume and randomisation of voxel inclusion at the contour edges. Image and mask perturbation methods were proposed by Zwanenburg et al.^12^ as a way to select robust features for modelling. These perturbations include small adaptations to the ROI mask, such as translation, rotation and randomised erosion and dilation at the edges. They concluded their perturbation approach was a viable alternative to feature comparison on test-retest imaging when this is not available.

The widely accepted standard for storage and handling of medical data is the “Digital Imaging and Communications in Medicine” (DICOM) format (https://www.dicomstandard.org). In a radiotherapy (RT) department, manual segmentations are drawn on top of an image by expert clinicians using specialised medical devices, including image contouring software and treatment planning systems. Within the vast DICOM standard, the Radiotherapy Structure Set (RTSTRUCT) module is the recognised format to store segmentation data for RT ROIs such as target volumes and organs at risk.

Contours in RTSTRUCT format are defined as 3-dimensional (3D) coordinate points (x, y, z) that represent closed polygon loops. The main data is stored in Contour Data attribute of the file (tag (3006, 0050)), and the attributes Image Position (tag (0020, 0032)) and Image Orientation (tag (0020, 0037)) of the associated DICOM images are vital to align contour and voxel data correctly. Image Position specifies the x, y, z coordinates of the upper left hand corner of the image and the Image Orientation specifies the direction cosines of the first row and column with respect to the patient, where axes direction are determined by the patient orientation. A full list of the mandatory modules and attributes recorded for RTSTRUCT can be found in^13^.

Most automatic segmentation methods inherently produce binary masks that are then converted to RTSTRUCT format to use in data communication and storage protocols within the hospital environment. Working with RTSTRUCT data collected from a clinical setting requires conversion of a polygon contour into a binary mask by determining which voxels lie sufficiently within the enclosed polygon space. A binary mask representation of a segmentation is needed for traditional radiomic analysis. Hence, the need for conversion between the two representations.

The reliability of polygon to mask conversion strategies is a well-known problem in computational geometry^14^. The IBSI reference manual provides details on a common technique to determine whether a point lies within a 2D (2 dimensional) polygon known as the crossing number algorithm^7^, alongside a description of a naïve implementation. However, there are many techniques that could be used^14^ and no one method was selected or suggested by the IBSI. Particularly with commercial products, the underlying mask generation method is often obscured from the end user. Furthermore, strategies such as super-sampling are used to fine tune selection of region voxels. The many different parameter settings for super-sampling strategies result in slight variations in the final mask.

In the radiomics reporting guidelines of the IBSI^7^, and a subsequent checklist by Pfaehler et al.^6^, it is explicitly suggested to describe the method used to convert polygon segmentations to a binary mask. However, this remains rarely reported in practice^6^. Knowledge of the polygon to binary mask conversion algorithm is necessary to ensure consistency and interoperability of radiomic features extracted from imaging and contouring data processed with different software applications. As DICOM is non-proprietary industry standard for data exchange—and most used file structure for biomedical images and metadata including contouring data—it is necessary to understand the full impact that discrepancies in binary mask generation could have on reportedly standardised features, particularly when radiomics algorithms are integrated in different medical image processing software platforms.

Despite an abundance of studies evaluating the robustness of radiomics to manual and automatic segmentation, to the best of our knowledge, no study has yet properly assessed standardised radiomic feature sensitivity to underlying mask generation algorithms, which still differ across both commercial and research-based medical imaging software.

The second aim was to determine whether mask generation alone could have a meaningful impact on quantitative analysis by examining the changes to raw feature values and to patient clustering, which in turn can influence radiomics based models.

## Study design

### Overview

**Figure 1:**
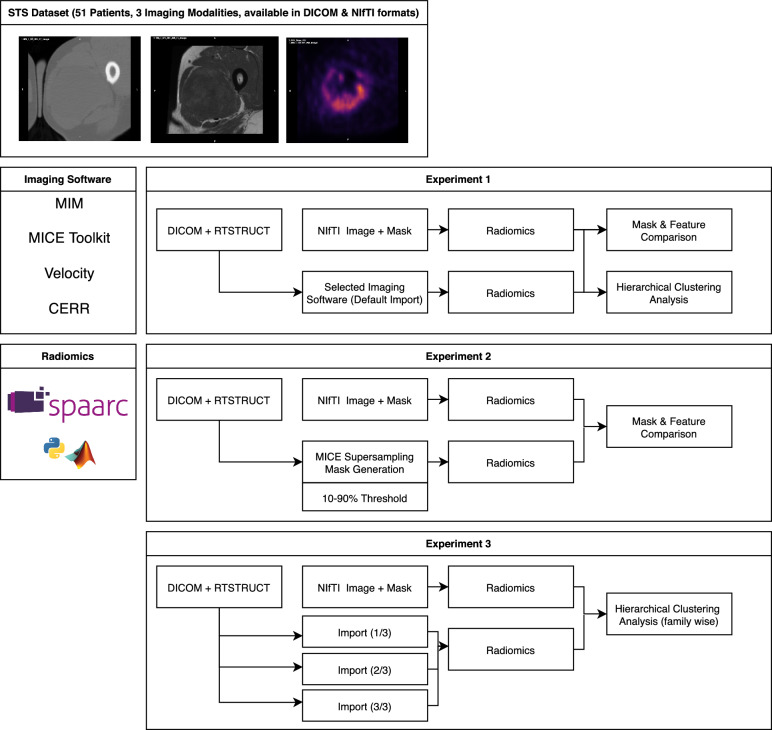
Study overview. This work used an open dataset of 51 patients with soft-tissue sarcoma (STS) (all patients had CT, MRI, and PET imaging). Original data was provided in both DICOM + RTSTRUCT and NIfTI formats. The study was split into 3 experiments. Experiment 1: DICOM data was imported with 4 software: MIM, CERR, MICE Toolkit, and Velocity. Resulting mask generation and feature extraction results were compared to a baseline extraction using the NIfTI files. Experiment 2: mask generation with super-sampling was evaluated using MICE Toolkit structure processor with different voxel acceptance thresholds. Feature values were again compared to baseline. Experiment 3: the dataset was split into 3 and imported using different mask generation techniques. Dataset was then recombined for radiomics and feature family-wise clustering compared to baseline. This experiment aimed to simulate a potential multi-centre radiomics collection.

The study workflow is shown in Fig. 1. The sensitivity of standardised radiomic algorithms to mask generation from DICOM RTSTRUCT was tested with 3 main experiments.

#### Experiment 1

We imported the DICOM dataset into four different imaging platforms (described in “Methods” section) with the default settings for each application. Using the API of each software, we passed the generated image, mask and voxel resolution to SPAARC for radiomic feature extraction. Correspondingly, we calculated features directly on the NIfTI version of the dataset, which were considered the baseline results.

The raw feature values calculated from the output of each imaging software were compared pairwise with the baseline using percentage difference and Spearman rank correlation $\rho$. We then assessed changes in hierarchical clustering of patients from choice of import software alone.

#### Experiment 2

**Figure 2:**
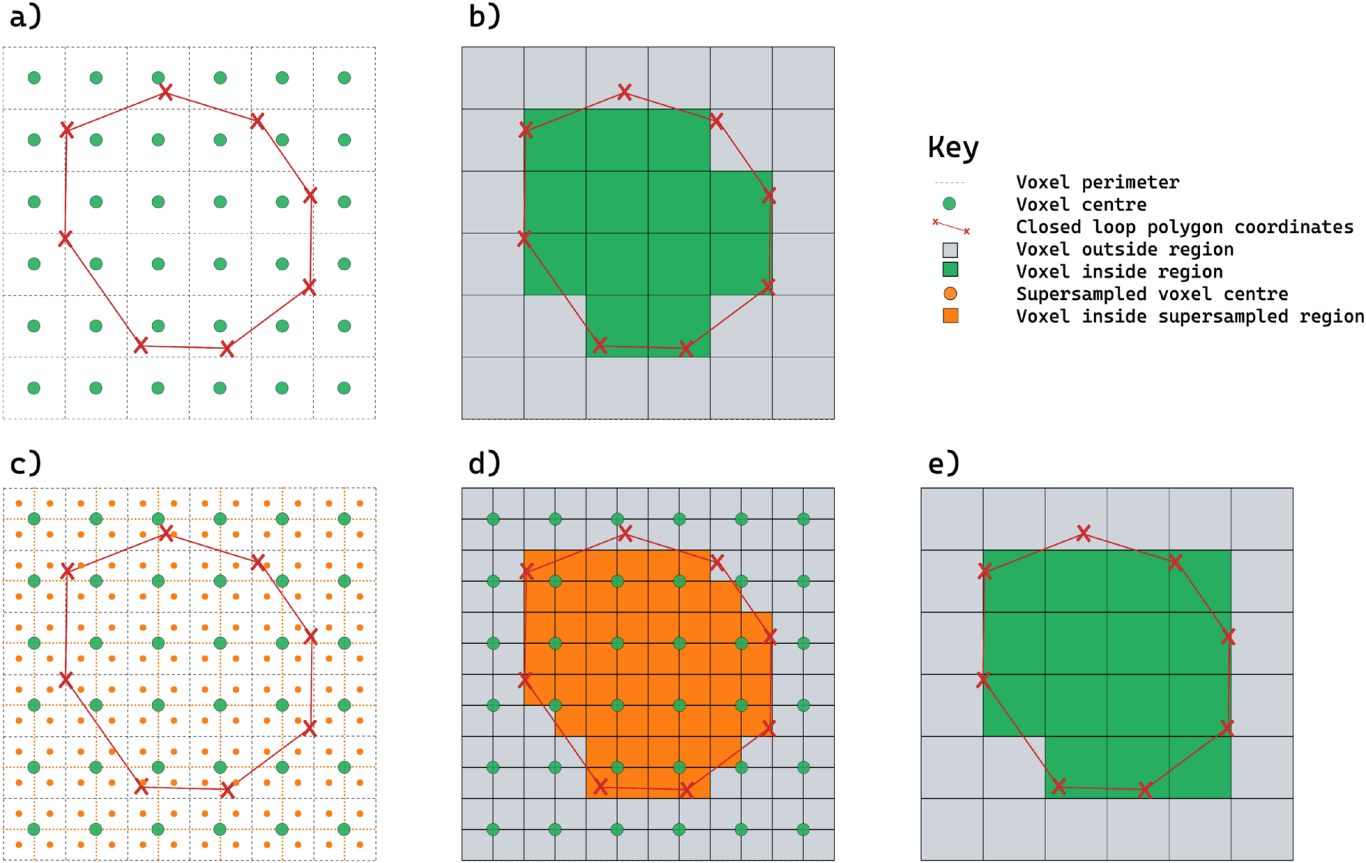
Illustrating conversion of coordinate points that define a closed polygon to a binary mask. (**a**) The coordinate positions of voxel centres are shown in green circles, and the perimeter of each voxel highlighted with a grey dashed line. Closed polygon loop shown with connected red crosses. (**b**) The resulting voxels that are within the region mask (green block). Here, if the centre of the voxel is within the closed polygon the voxel is considered part of the region. (**c**) Demonstrates new voxel centres (orange circles) after super-sampling the mask matrix in (**a**) by splitting each voxel down the centre in both axes. (**d**) The resulting super-sampled voxel centres that are within the closed polygon are highlighted in orange. (**e**) The resulting mask generated on the original dimension of (**a**), with a criteria that at least 25% of super-sampled voxels are within the closed loop polygon for a voxel to be within the region (in this case 1 sub-voxel). Note this leads to differences in the mask, comparing (**b**) and (**e**).

Additionally, we used functionalities available in MICE Toolkit to test the impact of mask generation with super-sampling using different voxel inclusion thresholds. This experiment aimed to systematically assess how voxel inclusion and exclusion at the mask edge impacts quantitative radiomics values. These were adjusted using settings in the MICE Structure Processor node. With this grid super-sampling method, each voxel is divided into sub-voxels. Whether each sub-voxel centre is within the closed polygon is then determined. For a voxel to be included, a threshold is set to define the percentage of sub-voxels within that voxel that are within the polygon. A comparison between the process of converting a of a polygon contour into a binary mask and the grid super-sampling method is shown in Fig. 2). In this study, the sub-voxel threshold was adjusted from 10 to 90%, in increments of 10%. Here we used a single split grid super-sampling that yielded 8 sub-voxels per voxel (i.e. each voxel is split down the centre of each axis).

#### Experiment 3

Finally, we split the dataset into 3 and imported with different mask generation settings before recombining into a single dataset. This is referred to here as the *mixed* dataset. The aim was to mimic collection and combination of radiomic data from 3 different centres where there was not a homogeneous approach to mask generation. We normalised the mixed and baseline feature extractions separately and compared hierarchical clustering differences at the feature family level.

The mixed import mask generation approaches were: (1) standard import (with CERR), (2) super-sampled import (with MICE—threshold 90%, grid split 1) (3) super-sampled import (with MICE—threshold 50%, grid split 3).

### Analysis

#### Mask difference

Mask differences produced by each import software were analysed pairwise with the corresponding baseline NIfTI mask. All masks for each patient were compared. For pairwise comparison a difference map ${\textbf{d}}_{map}$ was used to highlight the areas of discrepancy between masks. When comparing two mask matrices, ${\textbf{m}}_{a}$ and ${\textbf{m}}_{n}$, the difference map is simply the element-wise subtraction of one matrix from the other:

1 $$\begin{aligned} {\textbf{d}}_{map} = {\textbf{m}}_{a} - {\textbf{m}}_{n}. \end{aligned}$$

Voxels within the region in ${\textbf{m}}_{a}$ but not in ${\textbf{m}}_{n}$ correspond to a value of 1 in ${\textbf{d}}_{map}$. Conversely, voxels within the region in ${\textbf{m}}_{b}$ but not in ${\textbf{m}}_{a}$ correspond to -1 in the difference map. Voxels with the same assignment have a value of 0. Taking the absolute of ${\textbf{d}}_{map}$ and summing provided the voxel discrepancy number, $V_d$, which was scaled by the number of voxels in the ROI of the baseline, $N_m$ (in this case the NIfTI file), and stated as a percentage:

2 $$\begin{aligned} V_d = 100\times \frac{\sum _i \vert {\textbf{d}}_{map}^i\vert }{N_m}. \end{aligned}$$

**Figure 3:**
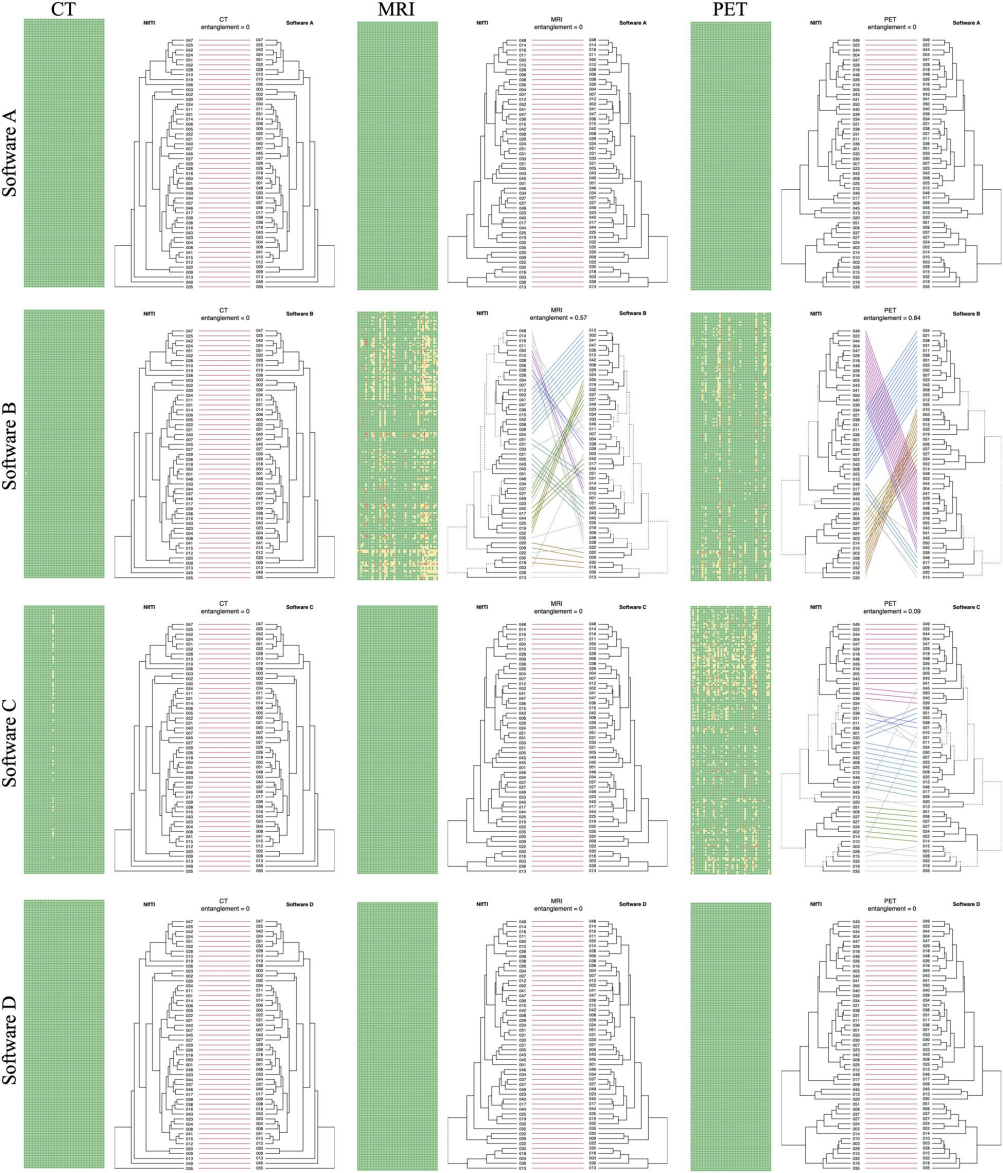
Measured differences in feature values and subsequent hierarchical clustering of patients when importing DICOM RTSTRUCT using 4 different imaging software. There is a heatmap and dendrogram comparison for each software, for each of the 3 modalities (CT, MRI, PET). Percentage difference between values was colour coded into 4 groups (green: < 0.5%, yellow: 0.5–3%, orange: 3–10%, and red: <10%). Hierarchical clustering using the baseline features was compared to the different software results (Left dendrogram: baseline clustering). Feature differences from mask generation at import can, alone, lead to significant unsupervised clustering differences.

#### Feature comparison

For each software import with DICOM, features were extracted using the SPAARC interface and compared pairwise with the baseline extraction set from NIfTI files with pre-defined ROI mask. The pairwise variation between respective raw feature values, (e.g., $f_A$ and $f_B$) was expressed as a percentage difference:

3 $$\begin{aligned} P_d = 100 \times \left| \frac{f_A - f_B}{(f_A + f_B)/2} \right| \end{aligned}$$

To avoid division by zero, any matching 0 value features were defined as having no variation explicitly. For visualisation, the measured difference, $P_d$, was used to categorise the result into 4 groups 0–<0.5%, 0.5–3%, 3–10%, and > 10%. The four groups were chosen to span a wide range of values, from 0 to infinity. The use of four groups provided enough granularity to distinguish between small and large differences, while still keeping the number of groups manageable. The cutoffs between the groups are were chose to represent thresholds of increasing significance. For example, the jump from [0.5–3%] to [3–10%] represents a three-fold increase in the magnitude of the difference being reported. We produced a heatmap for comparison. The groups had corresponding colors: green yellow, orange and red. The intraclass correlation coefficient (ICC) was used to assess feature consistency when extracted using different mask generation approaches. ICC values lie between 0 and 1 with a threshold > 0.9 often used to express high repeatability. With ICC we considered each mask generation as rater and each patient as subject. ICC was calculated using the Pingouin open-source statistical package^15^ written in Python 3 (Python Software Foundation, https://www.python.org). This implementation reports all the six cases of reliability of ratings described by Shrout and Fleiss^16^. Here we report ICC2, that considers a random sample of raters rating each target and that measures absolute agreement in the ratings. In addition for each feature, we assessed the stability of patient ranking using Spearman’s rank correlation coefficient ($\rho$) to investigate changes in the patients ranking which is important for the use of radiomic-based models.

#### Hierarchical clustering

With the feature data, we performed hierarchical clustering using R software (ver. 4.2.1, https://www.r-project.org) with the following packages: *cluster*^17^, and *dendextend*^18^. For each extraction, features were first scaled using Z-score. Hierarchical clustering was then performed using *complete-linkage* clustering and visualised using a dendrogram, which were compared using *dendextend*. Entanglement between two trees was measured (using *dendextend*) with L norm value set to 1. This is a gauge of cluster similarity between 1 and 0, where no entanglement (i.e. 0) is found for matching cluster ordering.

### Experiment 1

Differences in feature values ($P_d$), between the 4 import software and the baseline extraction from NIfTI, is visualised in Fig. 3. For a given heatmap, each column corresponds to 1 of the 51 patients, and each row to one of 158 radiomic features with colour categories as outlined above. Patient ranking with $\rho$ was assessed for every feature, for each of the 3 modalities. Ranking remained highly consistent across all tests, despite measured mask discrepancies and raw feature value differences, with no result $\rho <0.9$ (see Fig. 13 in Supplementary Materials Section C). High level of consistency between features extracted using different mask generation approaches was also confirmed by the intraclass correlation coefficient analysis with no result $ICC<0.9$ (see Fig. 14 in Supplementary Materials Section D).

Figure 3 also visualises hierarchical clustering changes due to import software choice. The left dendrogram is the baseline clustering, which is visually compared to the dendrogram resulting from the same clustering performed on feature data from a given import software (A, B, C and D). It can be noted that small differences in raw feature values due to mask discrepancy has the potential to significantly affect unsupervised patient clustering. The comparison of dendrograms, referred to as a tanglegram^18^, highlights unique nodes with dashed lines that contain combinations not present in the other tree. Connected lines are coloured to show common sub-trees between the two clusters.

For the CT data import, mask generation and feature extraction remained highly consistent across all software compared to the baseline, with only 2 small mask discrepancies that had no effect on subsequent normalisation and hierarchical clustering of features at the cohort level. For the MRI data import, software B had 36/51 patients with voxel discrepancy ($V_d$) in the masks, which led to many small feature variations and a significant difference to hierarchical clustering (entanglement = 0.57). For the PET data import, both software B and C measured $V_d$ in 16/51 and 36/51 masks respectively, which also led to measurable feature variation and changes to hierarchical clustering. Detailed, full size versions of all heatmaps including specific feature tags can be found in the Supplementary Materials Section B.

**Figure 4:**
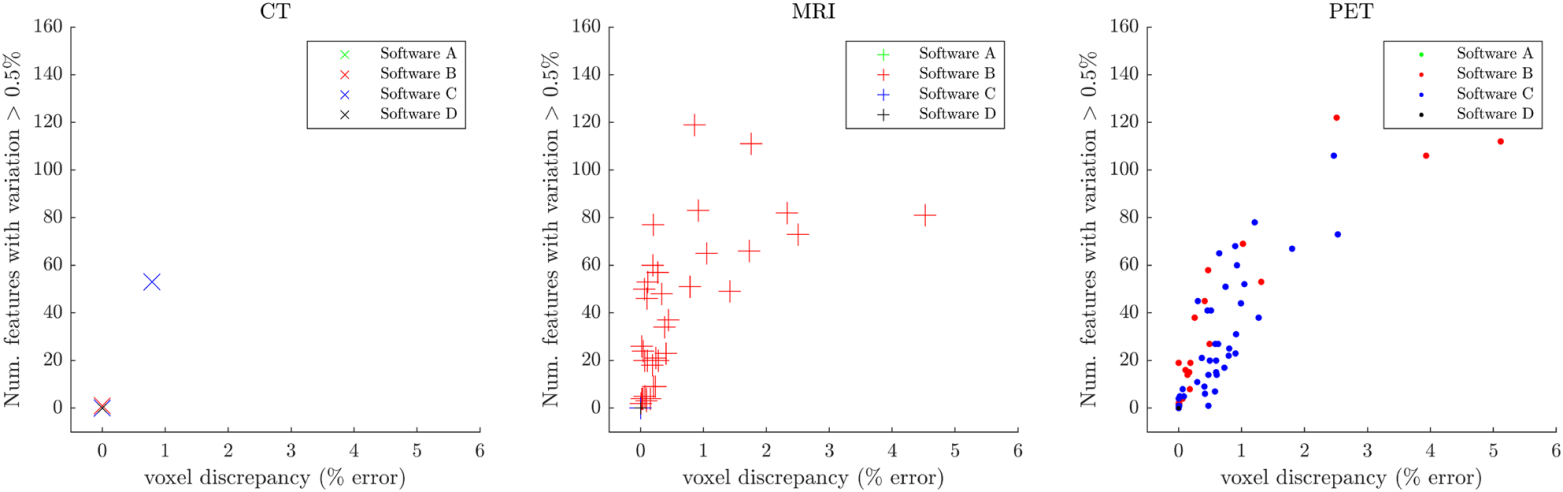
Voxel discrepancy in the mask ($V_d$) compared to number of features showing variation $P_d>0.5\%$. Each point corresponds to a patient. There is a separate plot for each imaging modality (CT, MRI, PET). Each software is colour-coded (green: A, red: B, blue: C, and black: D). As software A and D measured no mask discrepancy in any patients for any modality compared to the baseline, all points lie at the origin.

In general, the greater the $V_d$, the more features varied from the baseline result. This is illustrated in Fig. 4. For each patient, we plotted the number of features with $P_d>$ 0.5% against $V_d$, which yields a positive trend. It can be noted that many points lie at the origin, where masks exactly matched the baseline NIfTI version. Notably, this is the case for all patients from Software A and D. Two main types of mask discrepancy were observed. (1) Discrepancy occurred at the superior and inferior planes of the masks, resulting in an additional slice in one of the masks. (2) Alternatively discrepancies happened throughout the slices at points along the edge of the region.

**Figure 5:**
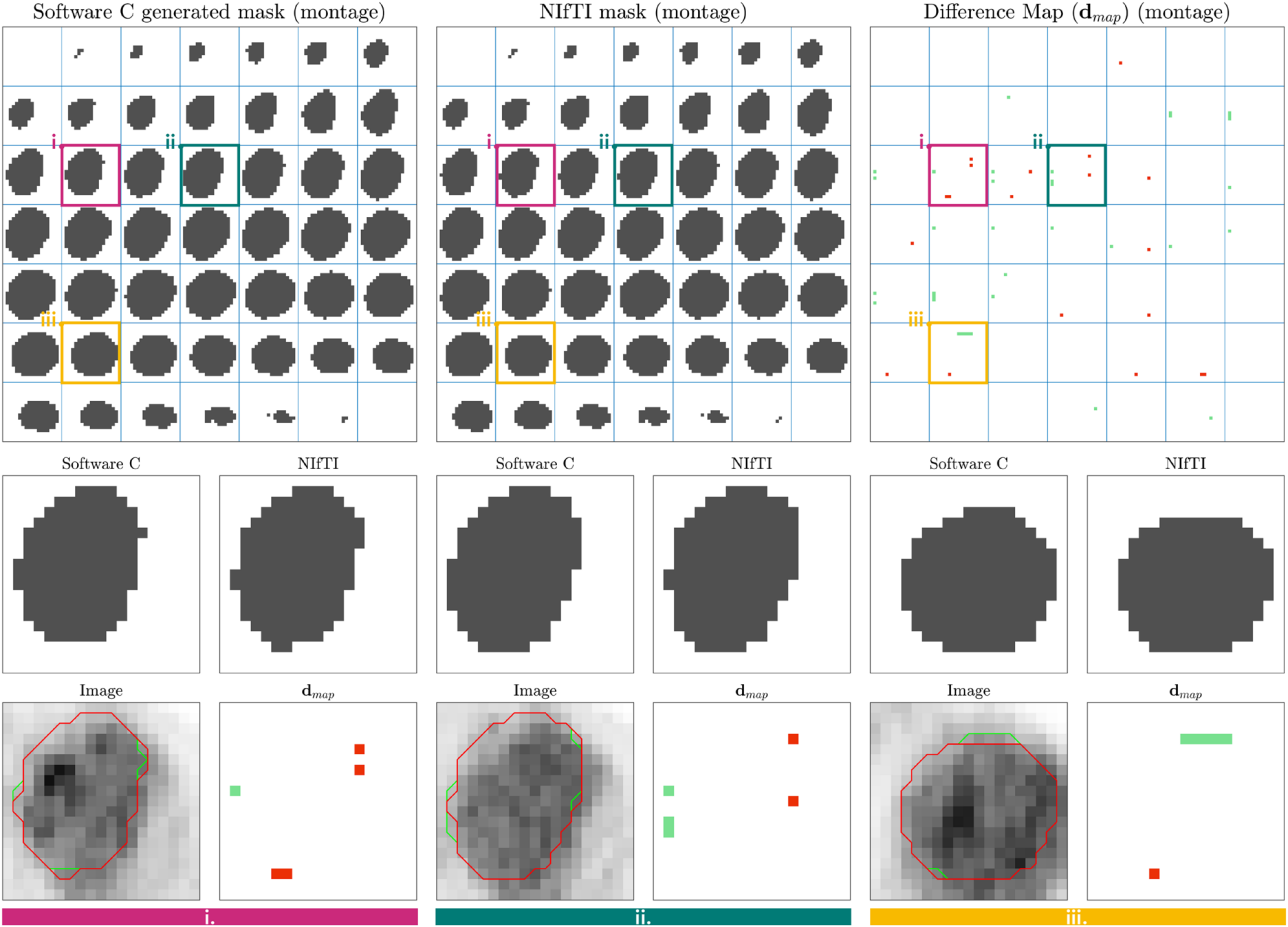
Representative example of mask discrepancy when importing with different software (Patient 5, PET). DICOM RTSTRUCT was converted to mask by Software C and compared to the corresponding NIfTI. The top row are montages of all mask slices (cropped for visualisation). Top right: difference map ${\textbf{d}}_{map}$ (red: voxel in NIfTI mask and not in generated mask; green: voxel in generated mask and not NIfTI mask). Three slices (i., ii, and iii.) were highlighted on the montages with coloured borders and a zoomed comparison provided in the bottom two rows, alongside the PET image for that slice.

In Fig. 5, we visualise a representative example of mask discrepancy for a PET imaging case. The mask generated by Software C is compared to the corresponding mask provided as a NIfTI. A montage of all relevant mask slices are compared. The difference map (${\textbf{d}}_{map}$) is visualised along 3 selected slices in greater detail. In ${\textbf{d}}_{map}$, red indicates voxels that are not in the NIfTi mask, while green indicates voxels that are not in the mask generated by Software C. These discrepancies lead to differences in image voxel intensities which are then included for feature analysis.

### Experiment 2

**Figure 6:**
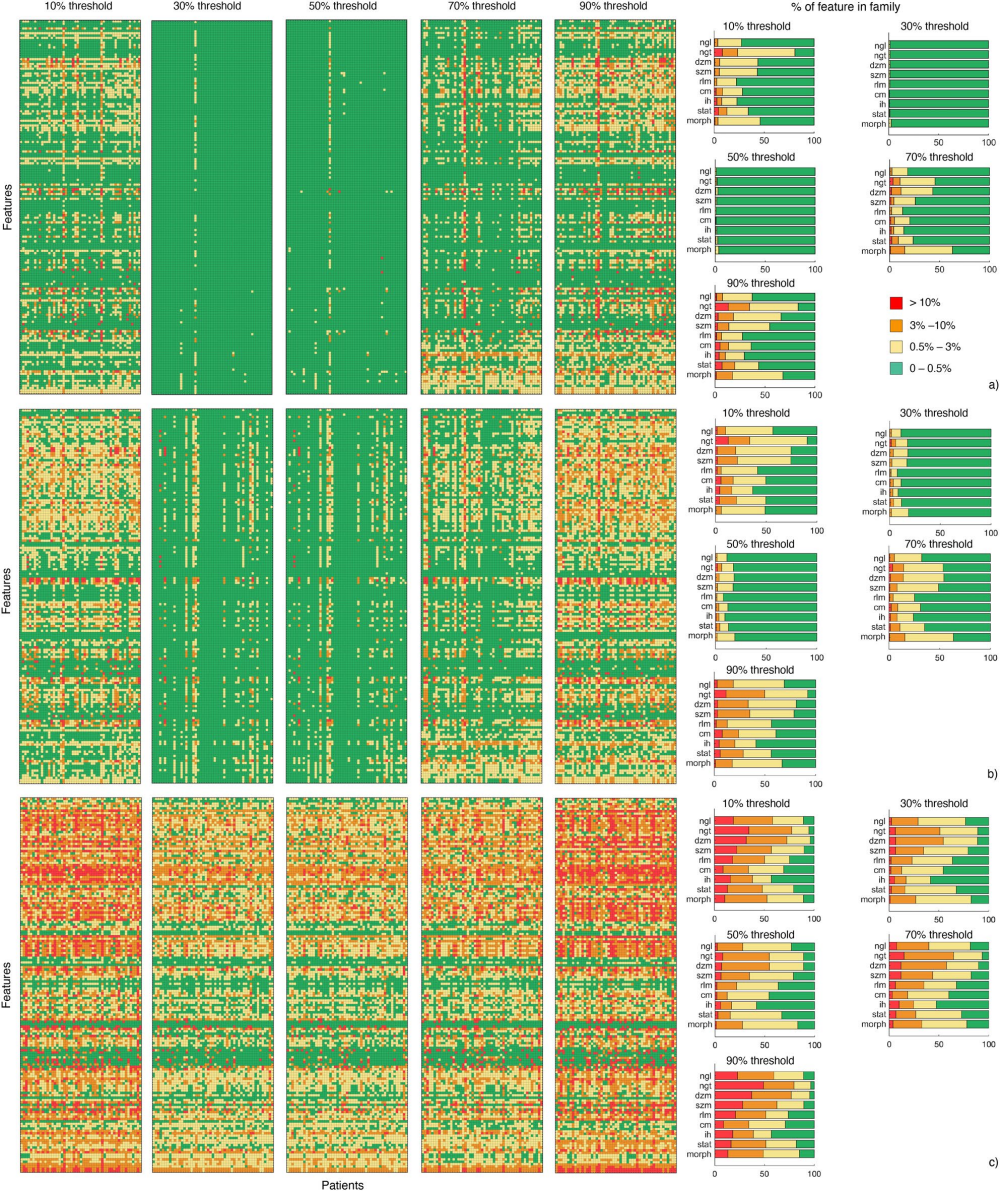
Overview of feature difference due mask super-sampling threshold settings. In each case, the features are compared to the corresponding extraction with the NIfTI format image and mask. There is a heatmap quantifying feature differences (15 in total) for CT (**a**), MR (**b**), PET (**c**) and for a grid super-sampling threshold setting: 10%, 30%, 50%, 70% and 90% . For a heatmap, each column corresponds to 1 of 51 patients, and each row corresponds to a feature. Features were categorised into 4 groups (0–0.5%: green, 0.5–3%: yellow, 3–10%: orange, and > 10%: red) based on percentage difference with the baseline extraction. Stacked bar charts summarise % variations divided into the 9 feature families.

Feature discrepancy due to mask generation with super-sampling is visualised with a range of heatmaps in Fig. 6. We report 5 heatmaps for each modality: CT (Figure 6a), MR (Fig. 6b) and PET (Fig. 6c). Results from each super-sampled voxel inclusion threshold (10–90% in steps of 20%) were compared to equivalent radiomic analysis extracted using the NIfTI files. It can be noted that the raw values for many features vary significantly ($P_d >10$%) when using a super-sampling strategy. The threshold chosen has a clear systematic influence on the features and masks, with more features changing as the threshold setting moves from a 50% sub-voxel inclusion. The use of stricter or more lenient thresholds (e.g. 90% or 10% respectively) leads to erosion or dilation effects at the mask boundary. This results in large numbers of voxels being included or excluded. We demonstrate this effect for PET in Supplementary Materials Section E. Mask differences due to super-sampling had a particularly pronounced effect on features in the PET imaging, as they contain fewer voxels within the ROI on average because they are lower resolution. Notably, in this particular case some threshold settings yield very similar or identical feature difference results (e.g. heatmaps corresponding to 30% and 50% settings in Fig. 6c), because very similar or identical masks were generated.

### Experiment 3

**Figure 7:**
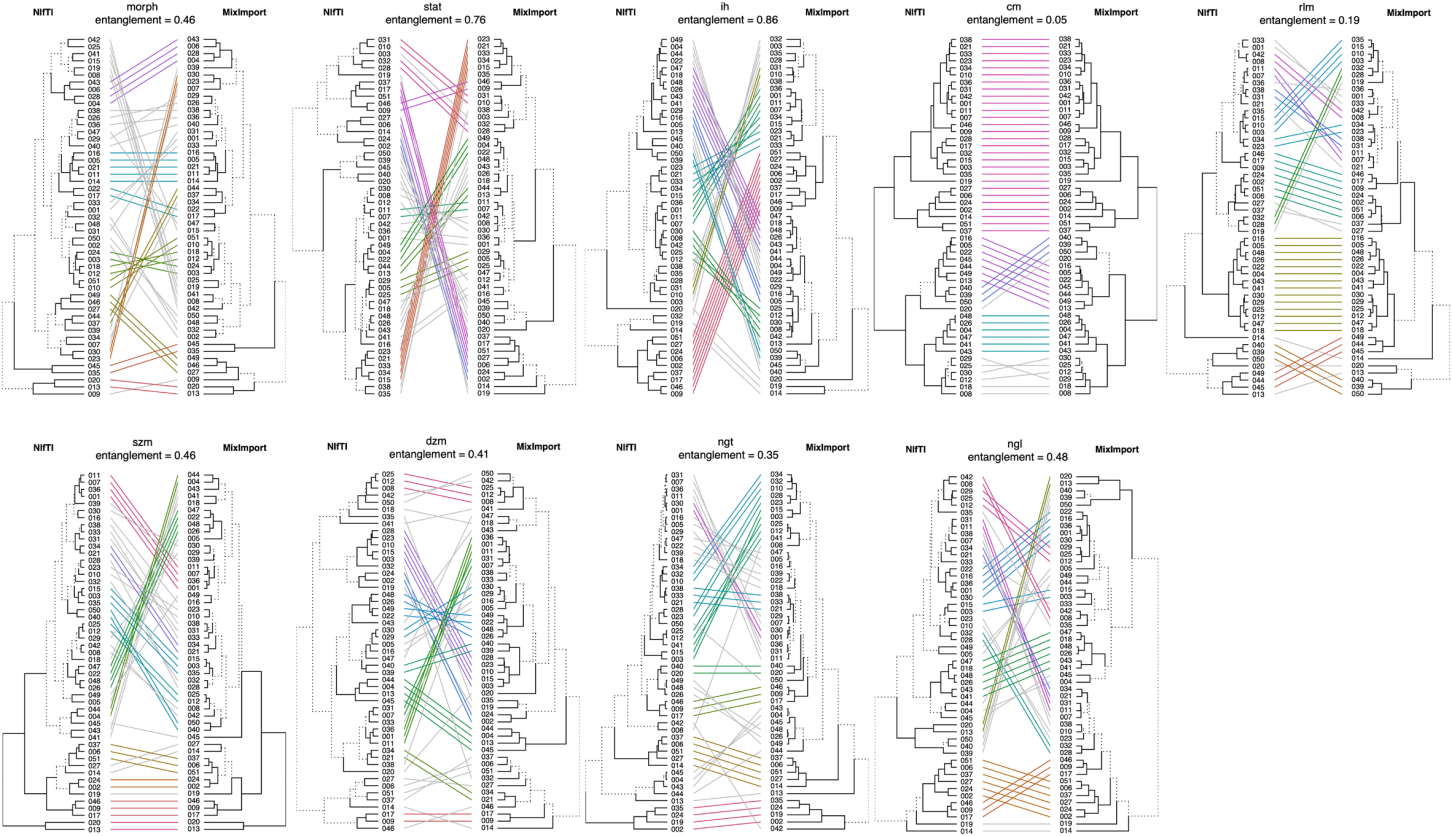
Comparison of hierarchical clustering of patients for different families of features, using the mixed dataset (combining different import strategies) compared to the baseline. The 9 feature families: Morphology (morph), Intensity based Statistics (stat), Intensity Histogram (ih), Gray Level Co-occurrence Matrix (cm), Gray Level Run Length Matrix (rlm), Gray Level Size Zone Matrix (szm), Gray Level Distance Zone Matrix (dzm), Neighbouring Gray Tone Difference Matrix (ngt), and Neighbourhood Gray Level Dependence Matrix (ngl). Hierarchical clustering using the baseline features (left dendrogram) was compared to the mixed results (right dendrogram), for PET. Clustering was greatly impacted by not having a consistent mask generation strategy, compared to the baseline data.

Using the mixed import dataset, changes in hierarchical clustering were compared at the family level for all the 3 imaging modalities. The results are shown in Fig. 7 for PET where the impact of mask generation algorithms were found to be higher. The discrepancy and entanglement of the family clusters make it clear that a mixed import with different mask generation strategies can be enough to severely effect the patient groupings based on their radiomic feature values, even though the underlying data remains the same. The results for CT and MRI are reported in Supplementary Materials Section F.

With clustering, features are normalised against other patients in their cohort, the effect of raw feature differences in some patient caused by mask discrepancy is thus distributed across the normalised feature values of the cohort. If mask discrepancy occurs in patients with outlier feature values, this can have a greater impact. This is an important consideration, as the underlying segmentation data is not different here (e.g. a different automated method or from a different clinician). This is just an effect of the chosen import software’s mask generation strategy. In multi-center studies where the import strategy may not be homogeneous, this variability should be a concern. Robustness strategies that remove features overly sensitive to slight variations of the mask boundary are a clear necessity.

## Discussion

This study assessed the sensitivity of standardised radiomics algorithms to binary mask conversion from DICOM RTSTRUCT data using different imaging software. We integrated our standardised radiomics platform with four imaging software to access their internal representation of volumes and structures to calculate imaging features. We preliminarily tested all four software against IBSI reference values. All four produced images and masks that led to features that are rigorously compliant to the benchmarks. Thus, their integration with our tool can be thought of as *IBSI compliant*. Despite this, we show in this study the same raw imaging features are not always achieved with a larger collection of data, and this was due to mask discrepancy.

With the exception of one case, where 53 features were affected by 1% voxel discrepancy in the mask, CT mask conversion was consistent across all four software. This resulted in exact agreement in feature values. However, we found that MRI and PET masks varied from the baseline for some software (B and C). The magnitude of discrepancy in the mask is patient and ROI specific, and the form of discrepancy was not consistent across patients. In other words, converting polygon contours lead to mask differences in some cases, while in others masks were exactly the same. This is clearly visualised in Fig. 3, where many heatmap columns remain entirely green (matching masks), whilst others show feature variation due to mask difference. We found discrepancy can manifest as an extension of the mask, superiorly and/or inferiorly, or with voxels at the edge of the ROI throughout the volume (Fig. 5). However, two of the software tested (A and D) produced masks that matched completely with the baseline and showed no feature difference as a result.

This work has highlighted that the magnitude of feature difference due to mask discrepancy is also modality and volume dependent. PET imaging in particular showed greater raw value variation. In general, PET images contain fewer voxels due to the lower resolution. We found that smaller volumes or low resolution scans with less voxels are more affected if mask discrepancy occurs. This is intuitive, as each individual voxel constitutes a greater proportion of the total volume. A few voxels difference in a CT ROI containing several hundreds of voxels does not lead to large feature value change. However, small PET volume with lower resolution may only contain a few tens of voxels making the impact of voxel loss relatively higher on feature computation. It is worth noting that in this work images and masks were not resampled with interpolation and features were extracted from original image dimensions passed to the feature extraction software. This was done to focus purely on the mask generation discrepancy which is the gap in knowledge that this paper address.

In Experiment 1, we have shown that despite very high level of ICC between features extracted from software implementing different polygon to mask conversion algorithms, changes in hierarchical clustering can still occur due to import software choice. This is non-trivial and highlights that rankings, as well as outcomes, can be affected by various complex interactions not fully captured by measurements of reliability or consistency.

In Experiment 2, we further demonstrated systematically the effect of super-sampling mask generation on radiomic features. Inherently, masks are different when this strategy is used, and features are not stable under different hyper parameters. As such, we recommend that super-sampling and non super-sampling mask generation methods should not be combined in a single radiomics study. We provide clear evidence that a consistent mask conversion strategy is required for completely replicable results on the same data. This is notable in the context of large multi centre and federated learning studies where the mask conversion strategy may not be consistent across participating institutions, which we simulated in Experiment 3.

To address the issues documented in this work we recommend that the type of polygon to mask conversion algorithm implemented in medical image processing applications (for both clinical or research use) is published as part of the software documentation. This will help harmonising the mask generation task and enable the definition of selection criteria for the inclusion of software packages when designing future quantitative image analysis studies. In addition, the use of the DICOM-Segmentation (DICOM-SEG) standard which can include a binary mask as the encoded type of segmentation data, would be recommended to store/exchange contours, ensuring interoperability and compatibility between different systems and applications. At the heart of this issue, radiomics modelling should select imaging features that are not susceptible to small discrepancies in the mask. This work complements the findings in Zwanenburg et al.^12^, that recommended deliberate image and mask perturbation strategies as a form of robustness testing for radiomic features. The degree of mask discrepancy we measured from different software imports alone is important in the context of standardisation.

One of the limitations of the study is that we did not assess the impact on feature computation due to the different parameters of the features such as, for instance, the different values of angle and distance in the case of Gray Level Co-occurrence Matrix (GLCM). Furthermore, since this work focused on polygon to mask conversion, we did not investigate if the different physical or computational processes and parameters behind the generation of the imaging modalities could be related to the variations found. Another limitation is that we used a publicly available dataset that included only one type of cancer. However, the focus of this study was on polygon to mask conversion and we have demonstrated the importance of this issue using 4 different independent software implementations, 3 different APIs and 2 programming languages for the same baseline radiomics code applied to 3 different imaging modalities. While this work focused on the application of engineered (as know as handcrafted) radiomic features, future investigations could explore the impact that polygon to mask conversion could have on the learning process of automatic features such as those based on deep learning and determine whether neural networks can compensate for such variations.

In conclusion, we assessed the sensitivity of standardised radiomic feature to the process of converting contours to binary mask, which differ across both commercial and research-based medical imaging software. Our findings show that mask generation does have significant impact on raw feature values and on patient clustering.

## Methods

### Imaging data

For this study, we opted to use the dataset from the validation phase of the IBSI study^7^. This cohort contains 51 patients with soft-tissue sarcoma (STS) who underwent 3-types of volumetric imaging: computed tomography (CT), T1-weighted magnetic resonance imaging (MRI) and fluorine 18-fluorodeoxyglucose positron emission tomography ($^{18}$F-FDG PET). The dataset originates from Valliéres et al.^19^ and was made publicly available on the cancer imaging archive^20^. See their work for more details^21^. The pre-processed versions used by the IBSI were made available in an online GitHub repository, and importantly for this work, are provided in both DICOM and Neuroimaging Informatics Technology Initiative (NIfTI) formats (https://github.com/theibsi/data_sets).

### Imaging software

In order to test independent polygon to mask conversion algorithms, the STS DICOM imaging dataset was imported in a range of applications that satisfied the following requirements: (1) available in either research or clinical domain, (2) compliant with the DICOM standard including the RTSTRUCT modality, (3) providing a suitable application programming interface (API) that could allow internal data (images and binary masks) to be accessible by third party applications such as our quantitative image analysis software. The following medical imaging applications were used in this study: (a) computational environment for radiological research (CERR) (commit 278f008): an open-source, Matlab-based platform for working with medical imaging data. CERR data import functionality creates an extensible and accessible data structure in a .mat file, convenient for prototyping algorithms and conducting research in medical imaging within the Matlab ecosystem^22,23^; (b) MIM (v7.1.5, MIM Software Inc., Beachwood, OH): a commercial imaging product with an optional MIM Extensions component, allowing users to develop extensions via a Matlab API, in which images and ROI structures can be passed from MIM to a Matlab environment for processing; (c) Medical Interactive Creative Environment (MICE) Toolkit (v2022.4.9, NONPI Medical AB) Toolkit: a graphical programming interface for performing image analysis using workflows. MICE enables creation of custom plugin nodes to run Matlab or Python on imaging imported into a MICE database; (d) Velocity (v4.1.2.1194, Varian Medical Systems, Palo Alto, CA) a commercial product with a focus on cancer imaging solutions. Velocity has a Python API called *VelocityEngine*^24^ that facilitates scripting over databases. This API was used to collect imaging and mask data from scans loaded into a designated workstation database. For reporting our experiments, in this manuscript we randomly anonymised the above into *Software A*, *Software B*, *Software C* and *Software D* labels.

### Feature extraction

Radiomic feature extraction was performed with Spaarc Pipeline for Automated Analysis and Radiomics Computing (SPAARC) (https://www.spaarc-radiomics.io) developed at our institution through active participation in the IBSI. From SPAARC we tested a total of 158 standardised features, including: 23 Morphology, 18 Intensity based Statistics, 23 Intensity Histogram, 25 Gray Level Co-occurrence Matrix, 16 Gray Level Run Length Matrix, 16 Gray Level Size Zone Matrix, 16 Gray Level Distance Zone Matrix, 5 Neighbouring Gray Tone Difference Matrix, and 16 Gray Level Dependence Matrix.

The SPAARC radiomics pipeline is implemented in 2 separate languages (MATLAB and Python), and both versions rigorously match the developed IBSI benchmarks for the features tested in this study. The MATLAB version of SPAARC (v1.8.1) was run in MATLAB 2021a. In addition, the MATLAB code natively supports the data format of CERR. The Python version of SPAARC was built in Python 3.9.10.

SPAARC requires 4 inputs: an image matrix, a mask matrix, image metadata (i.e., voxel resolution and image orientation), and a configuration file detailing the feature extraction settings. This is provided in the JavaScript Object Notation (JSON) open standard and language-independent and human-readable data interchange format. For each of the medical imaging software described in the Methods section, a plugin was written to access and make available to SPAARC the 4 inputs via the application’s specific API. The masks that were passed to SPAARC from each platform were also saved for further analysis and assessment of differences in the generation from RTSTRUCT.

For each of the 3 imaging modalities, the same 3 configuration files defined the settings used across the plugins. Furthermore, feature extraction was repeated directly on the available NIfTI versions of the data, which were then the baseline feature values used for this study. To focus purely on mask generation discrepancy, imaging and masks were not resampled with interpolation, so features were extracted from original image dimensions passed to software. The precise feature extraction settings used for each modality, and the configuration files, can be found in the Supplementary Materials Section A.

We preliminarily tested all four software integrations with SPAARC against IBSI reference values. These reference values use a CT lung phantom^7^. For each software, all benchmarks were met for the features tested in this work. Hence, these software integrations can be thought of as *IBSI compliant*.

### Supplementary Information

Supplementary Information.

## Data Availability

The datasets used during the current study are available in the The Cancer Imaging Archive: http://doi.org/10.7937/K9/TCIA.2015.7GO2GSKS. The datasets generated during the current study are available from the corresponding author on reasonable request.
