# Supplementary material for: Sensitivity of standardised radiomics algorithms to mask generation across different software platforms
Source: Sci Rep. 2023 Sep 2;13:14419. doi: 10.1038/s41598-023-41475-w (PMC10475062; doi:10.1038/s41598-023-41475-w)
Supplement: Supplementary file 1 — Supplementary Information. [file 41598_2023_41475_MOESM1_ESM.pdf]

Supplementary Materials:

'Sensitivity of standardised radiomics algorithms to mask generation across different software platforms'

Philip Whybra[1] and Emiliano Spezi*[1]

Corresponding author* (espezi@cardiff.ac.uk)

[1] School of Engineering, Cardiff University, United Kingdom

Contents

A Feature extraction configuration files **2**
A.1 CT . . . 2
A.2 MRI . . . 2
A.3 PET . . . 2

B Experiment 1: Heatmaps of measured feature variation **3**

C Spearman Rank **16**

D Intraclass correlation coefficient **16**

E Example of super-sampling mask effect **17**

F Hierarchical clustering **19**

G Extraction details **21**

Bibliography **25**

.

A Feature extraction configuration files

A.1 CT

```
{
  "feature_families": ["morph", "stats", "ih", "glcm", "glrlm",
                       "glszm", "gldzm", "ngldm", "ngtdm"],
  "re_segmentation_range": {"min": -1000, "max": ""},
  "re_segmentation_outlier_filtering": {"apply": false, "sigma": 3},
  "bin_method": "FBS",
  "bin_value": 10,
  "analysis_type": "3D",
  "texture_parameters":{
    "glcm": {"aggregation": "merged", "distance": 1},
    "glrlm": {"aggregation": "merged", "distance": 1},
    "ngtdm": {"distance": 1},
    "ngldm": {"distance": 1, "alpha": 0}
  }
}
```

A.2 MRI

```
{
  "feature_families": ["morph", "stats", "ih", "glcm", "glrlm",
                       "glszm", "gldzm", "ngldm", "ngtdm"],
  "re_segmentation_range": {"min": 0, "max": ""},
  "re_segmentation_outlier_filtering": {"apply": false, "sigma": 3},
  "bin_method": "FBS",
  "bin_value": 10,
  "analysis_type": "3D",
  "texture_parameters":{
    "glcm": {"aggregation": "merged", "distance": 1},
    "glrlm": {"aggregation": "merged", "distance": 1},
    "ngtdm": {"distance": 1},
    "ngldm": {"distance": 1, "alpha": 0}
  }
}
```

A.3 PET

```
{
  "feature_families": ["morph", "stats", "ih", "glcm", "glrlm",
                       "glszm", "gldzm", "ngldm", "ngtdm"],
  "re_segmentation_range": {"min": 0, "max": ""},
  "re_segmentation_outlier_filtering": {"apply": false, "sigma": 3},
  "bin_method": "FBS",
  "bin_value": 0.25,
  "analysis_type": "3D",
  "texture_parameters":{
    "glcm": {"aggregation": "merged", "distance": 1},
    "glrlm": {"aggregation": "merged", "distance": 1},
    "ngtdm": {"distance": 1},
    "ngldm": {"distance": 1, "alpha": 0}
  }
}
```

B Experiment 1: Heatmaps of measured feature variation

Full size versions of heatmaps shown in Figure 1 of the **main manuscript** (also shown below).

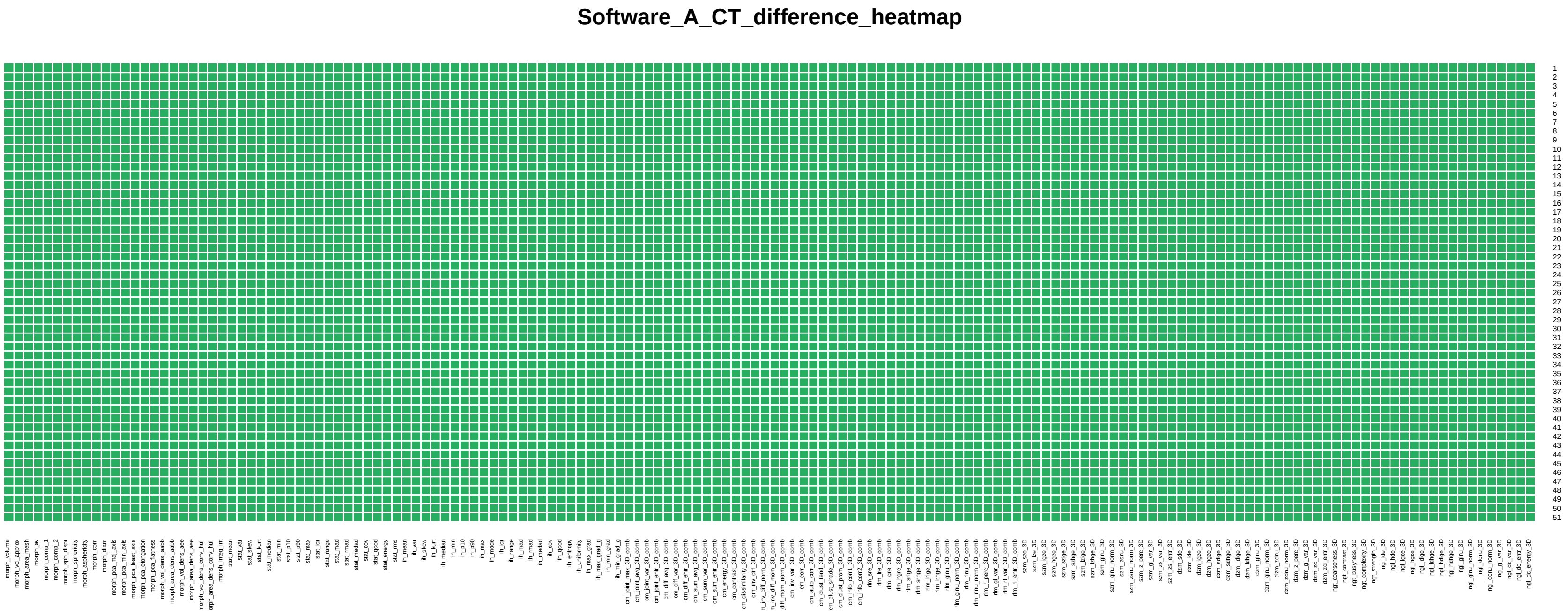

Figure 1: Software A compared to baseline (CT).

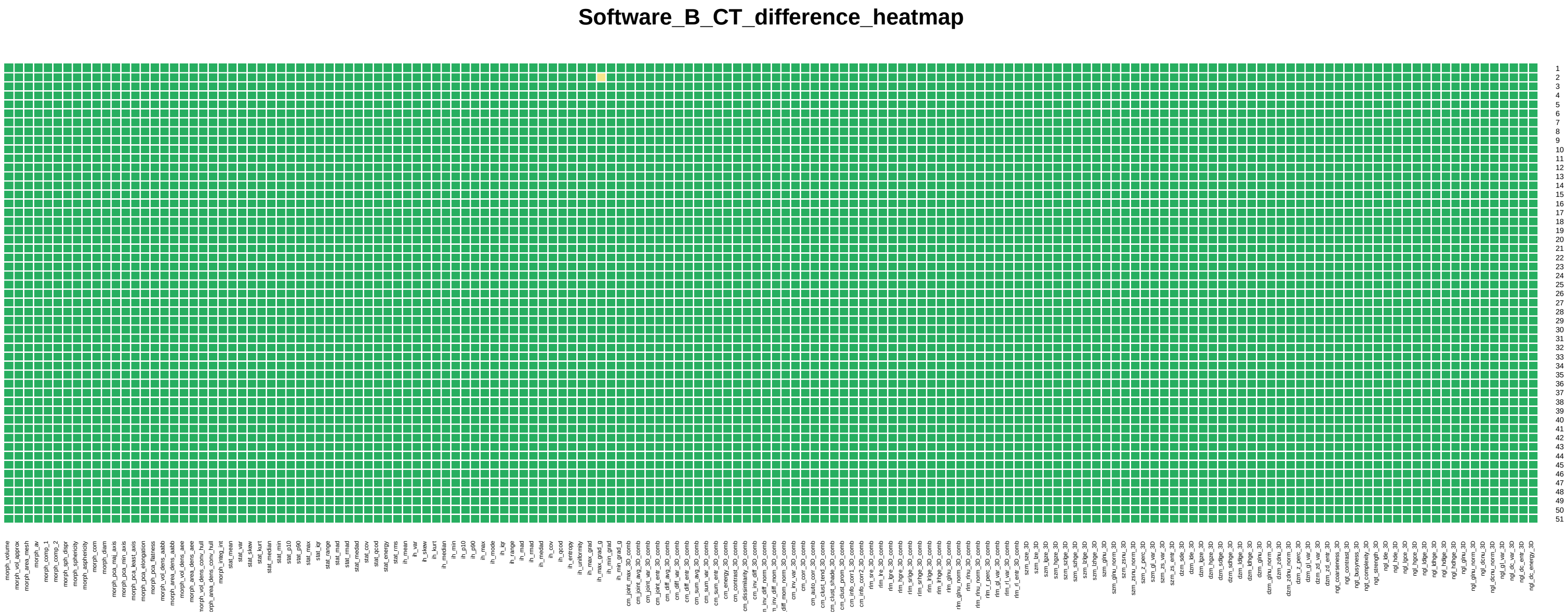

Figure 2: Software B compared to baseline (CT).

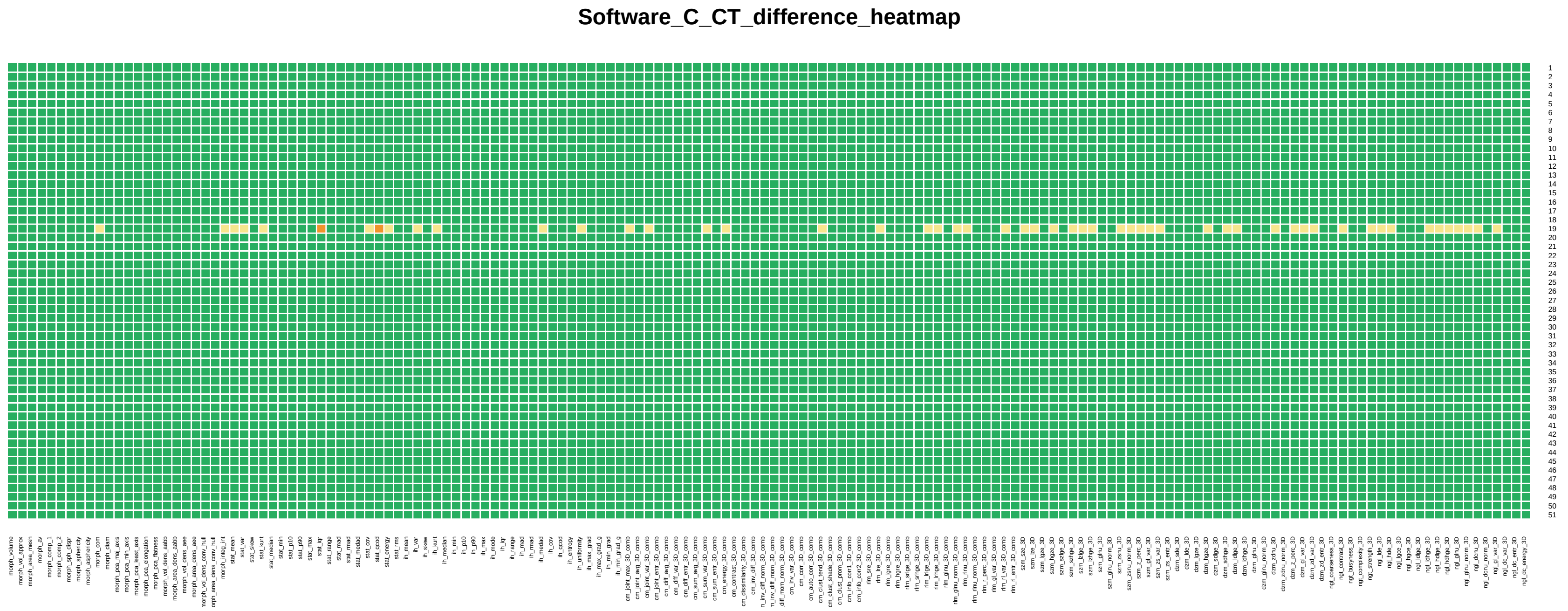

Figure 3: Software C compared to baseline (CT).

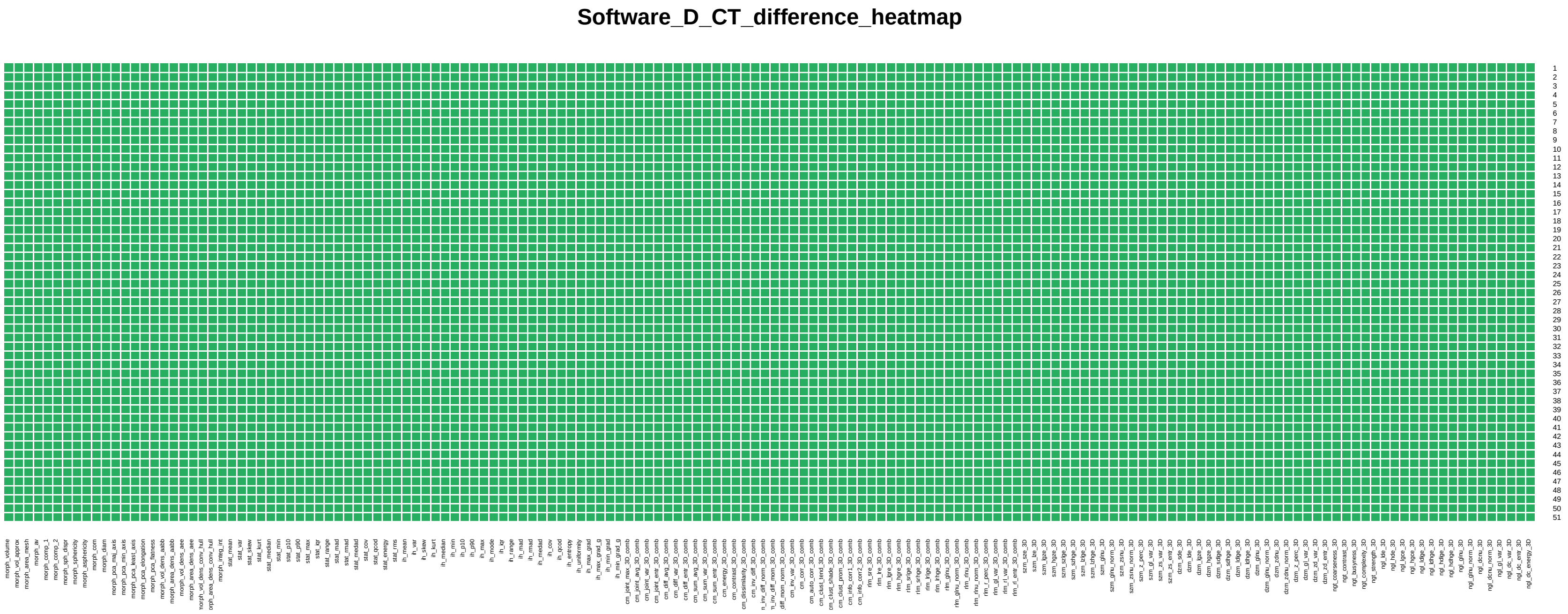

Figure 4: Software D compared to baseline (CT).

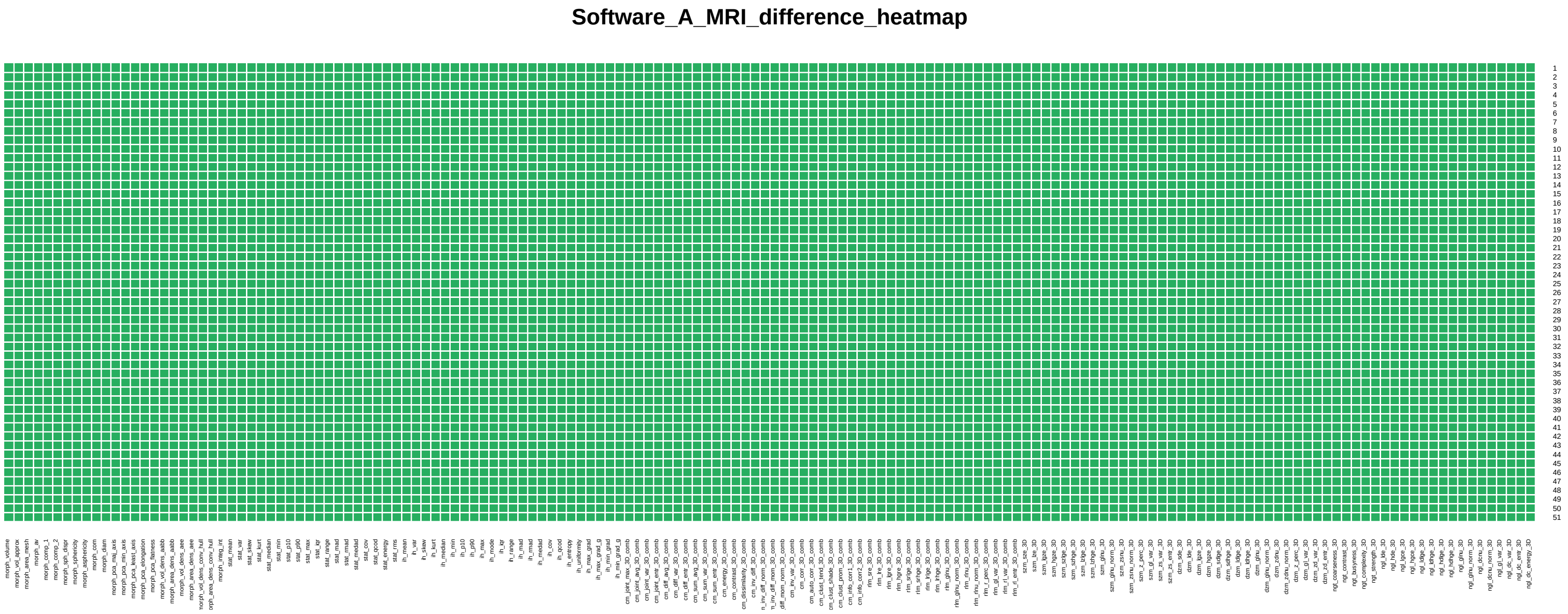

Figure 5: Software A compared to baseline (MRI).

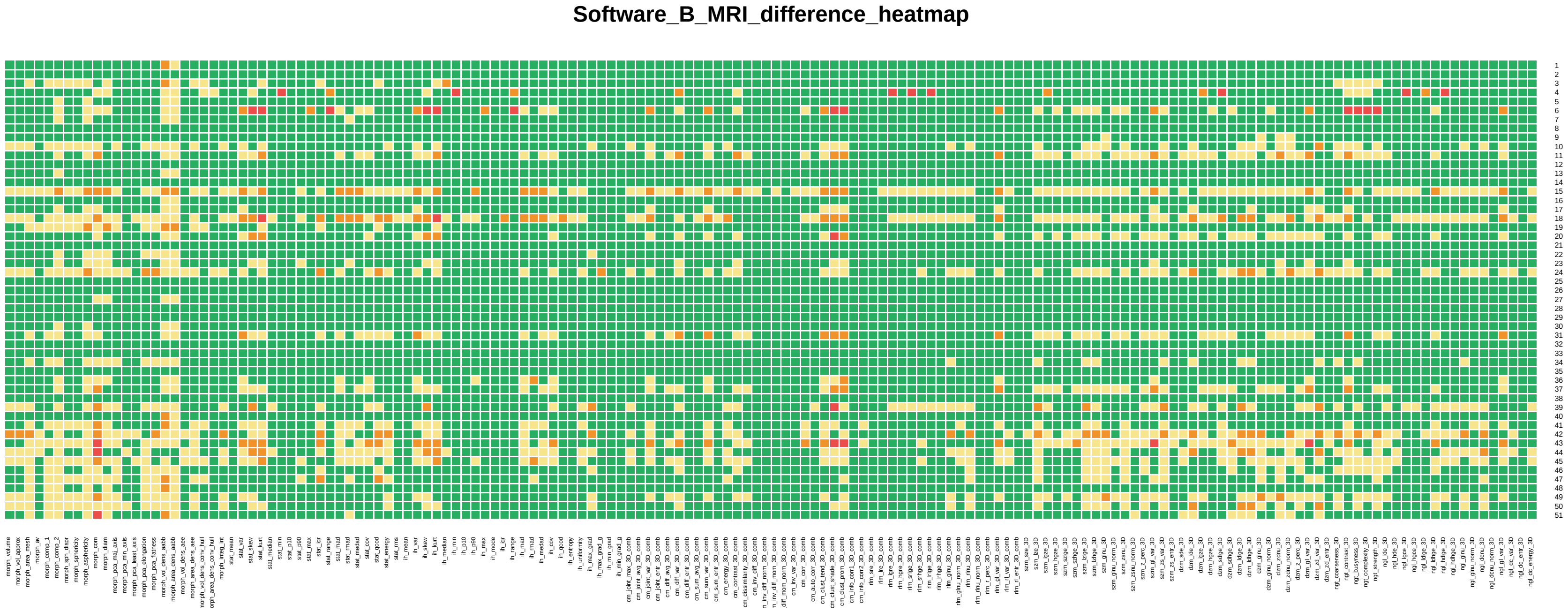

Figure 6: Software B compared to baseline (MRI).

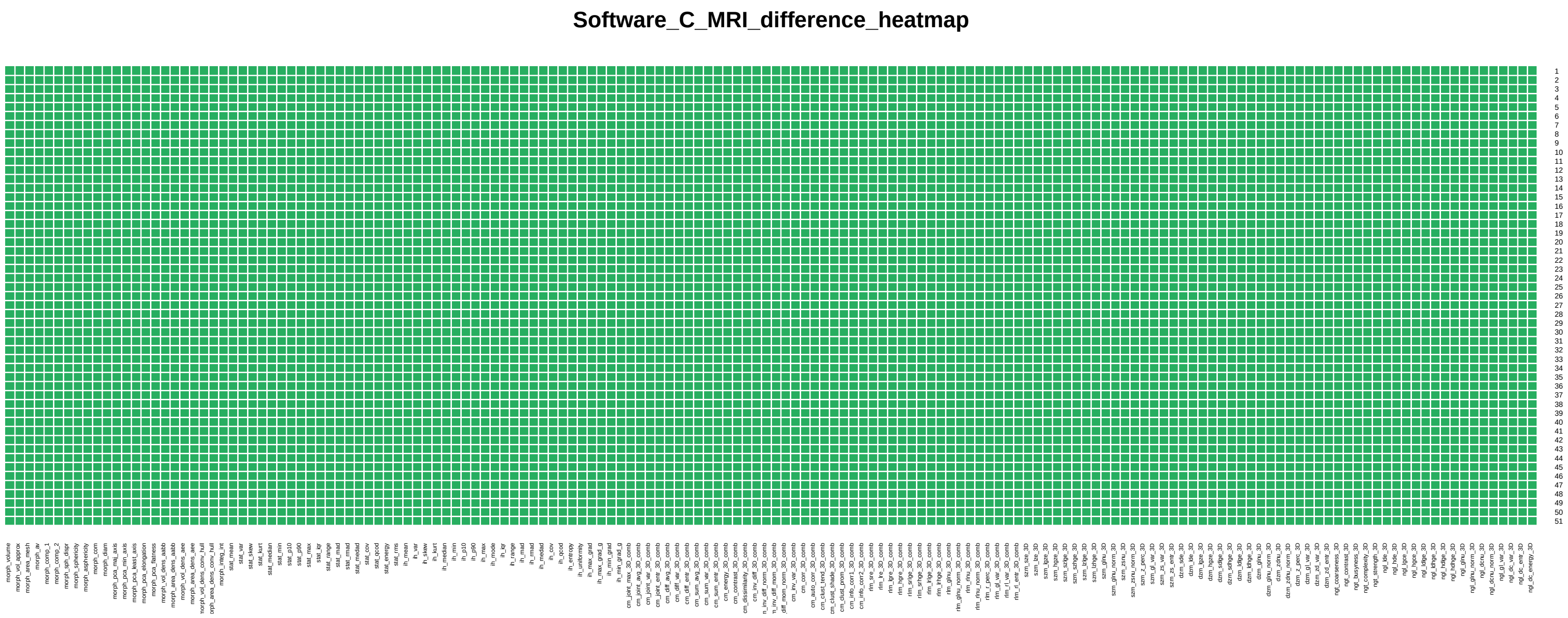

Figure 7: Software C compared to baseline (MRI).

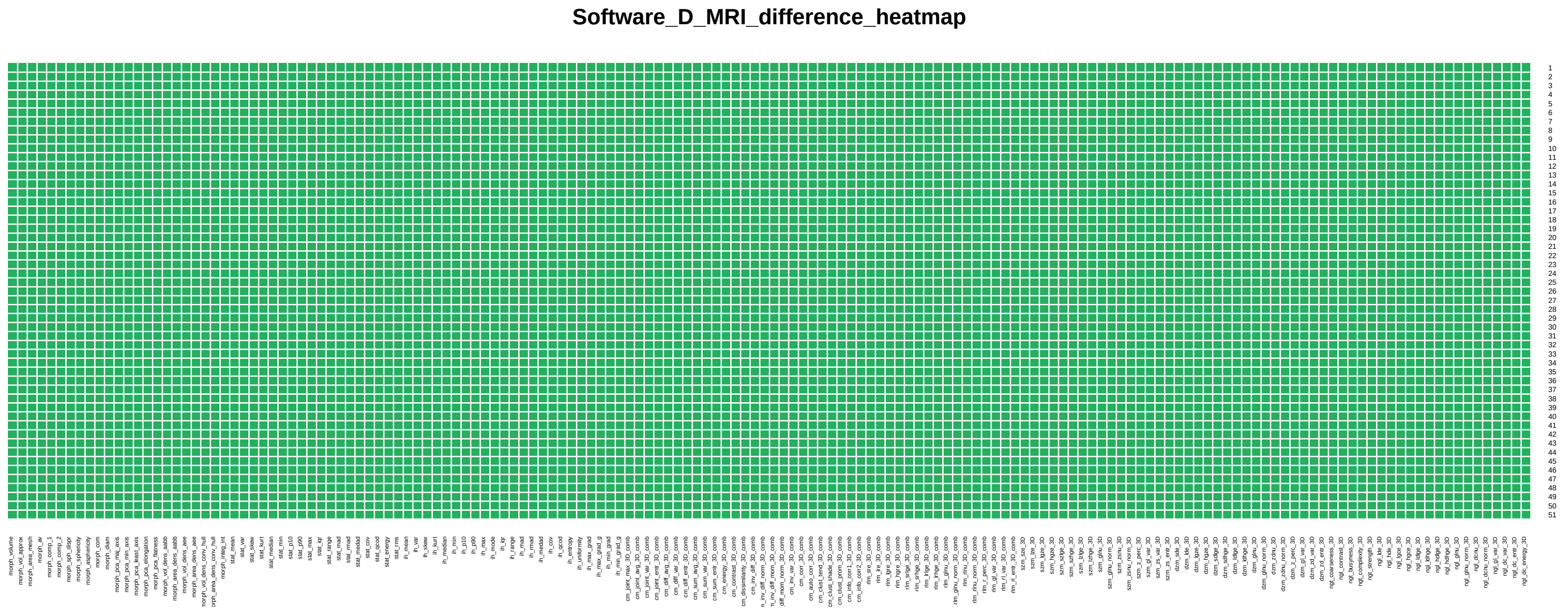

Figure 8: Software D compared to baseline (MRI).

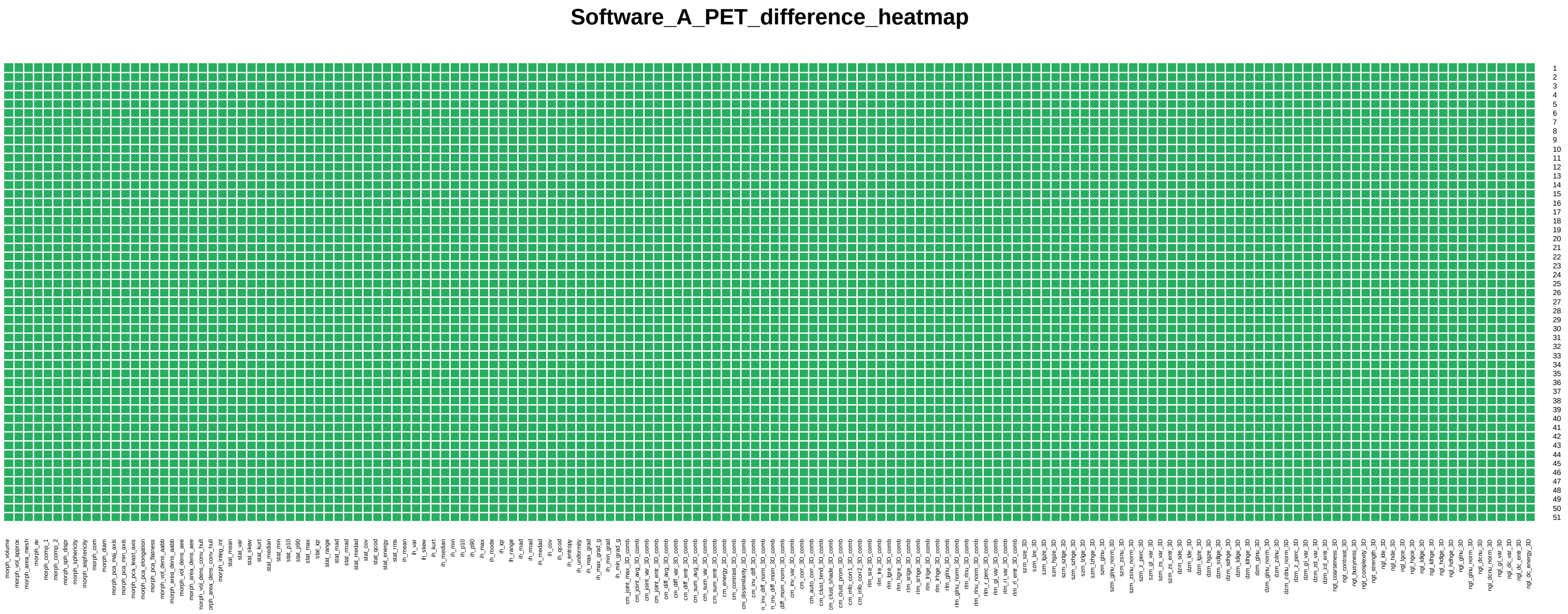

Figure 9: Software A compared to baseline (PET).

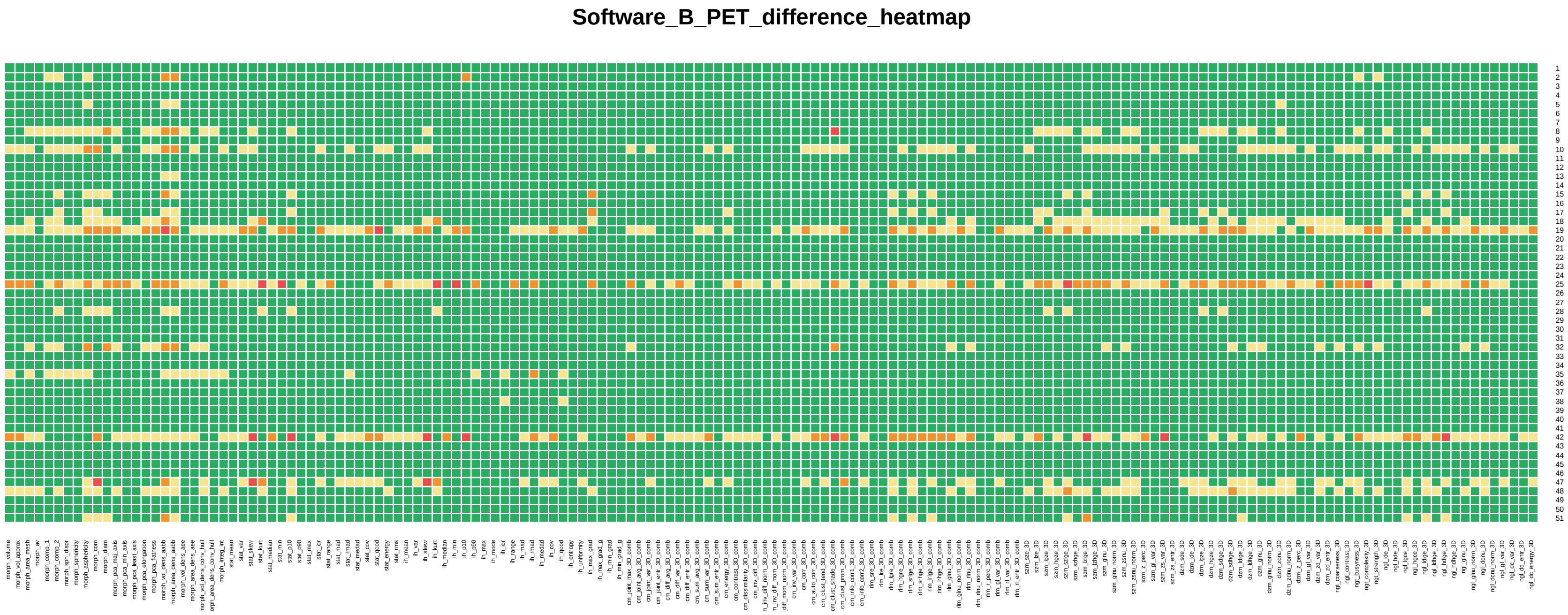

Figure 10: Software B compared to baseline (PET).

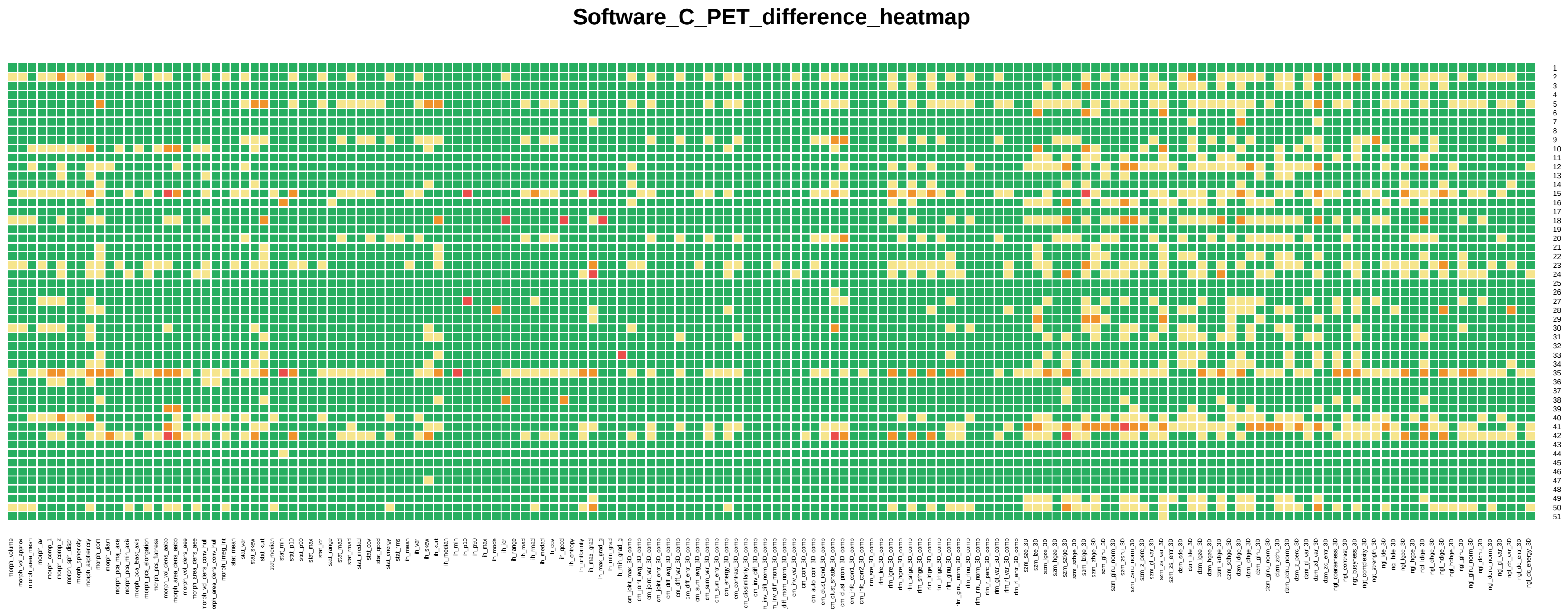

Figure 11: Software C compared to baseline (PET).

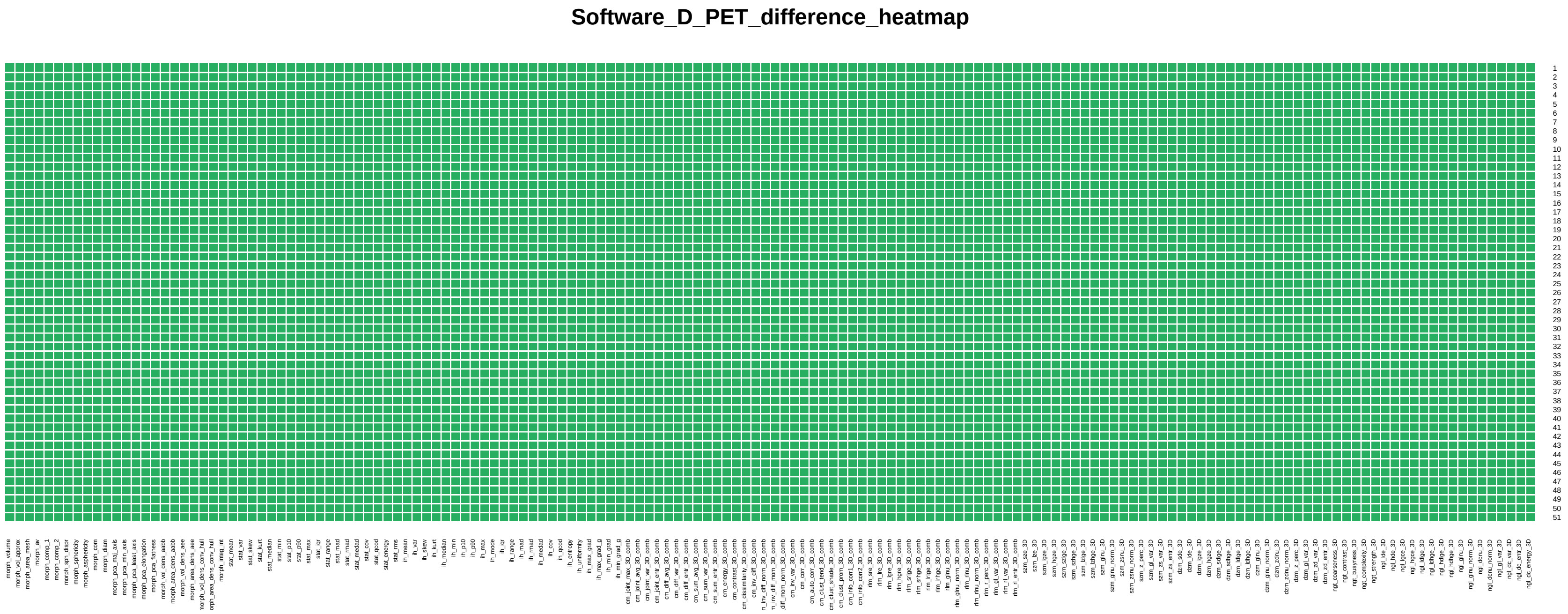

Figure 12: Software D compared to baseline (PET).

C Spearman Rank

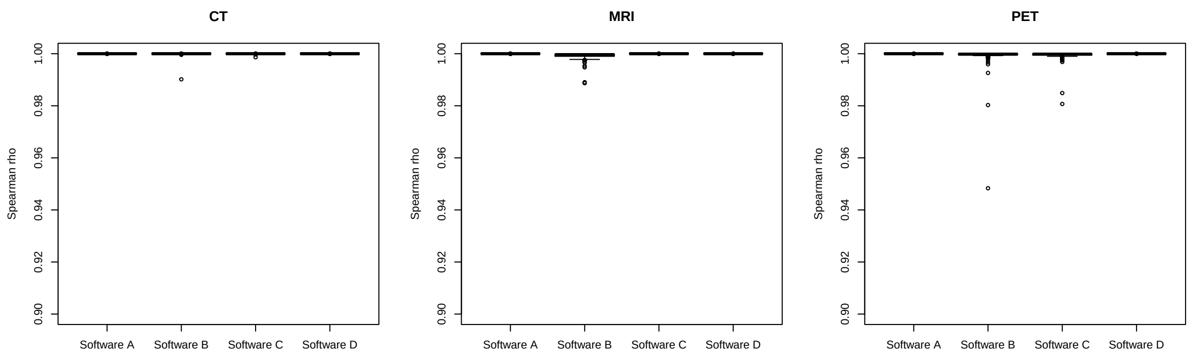

Figure 13: Box plots of Spearman rank correlation for all features when comparing standard import DICOM for each software, with using the NIfTI files. Evidently, all features remain highly correlated despite the measured mask discrepancy.

D Intraclass correlation coefficient

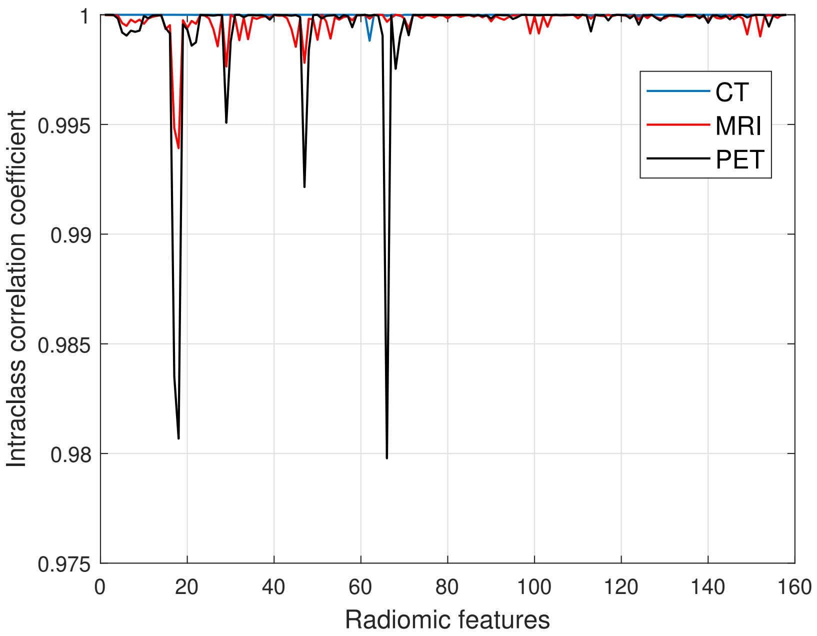

Figure 14: Intraclass correlation coefficient for software A, B, C and D, and for all the imaging modalities considered in this work. Despite a slight drop for PET, a very high level of concordance for all features is evident.

E Example of super-sampling mask effect

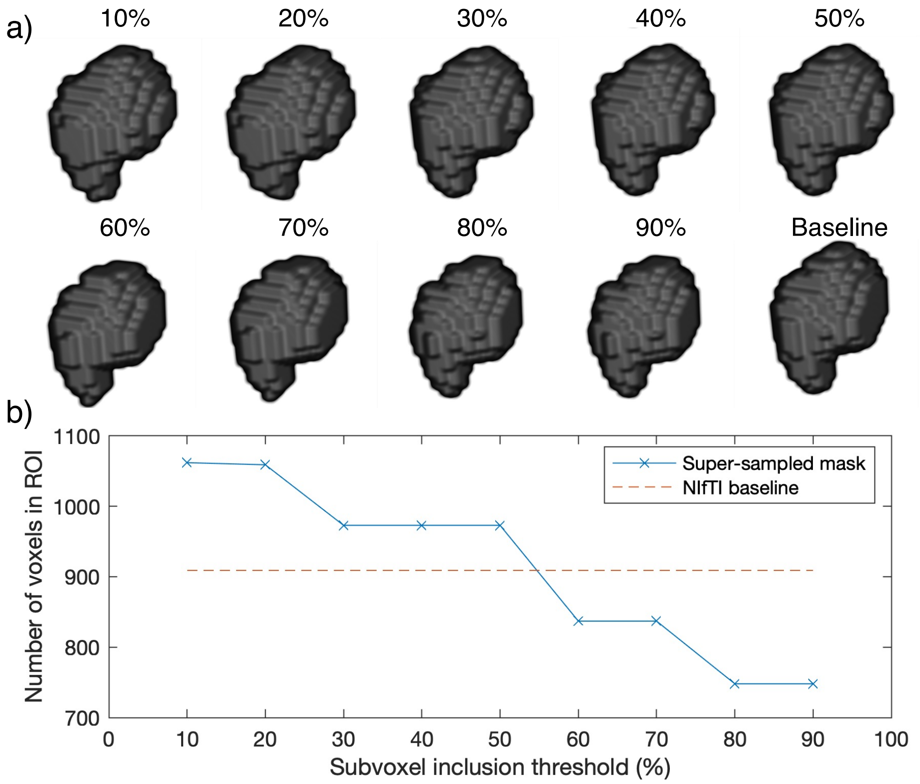

Figure 15: Effect of super-sampling thresholds on the mask (example PET ROI for STS case 15). As one increases the percentage of sub-voxels needed to be within the closed polygon (for the parent voxel to be included in the mask), we get a smaller volume. **a)** Visualisation. **b)** Subvoxel inclusion threshold (%) against number of resulting voxels in ROI.

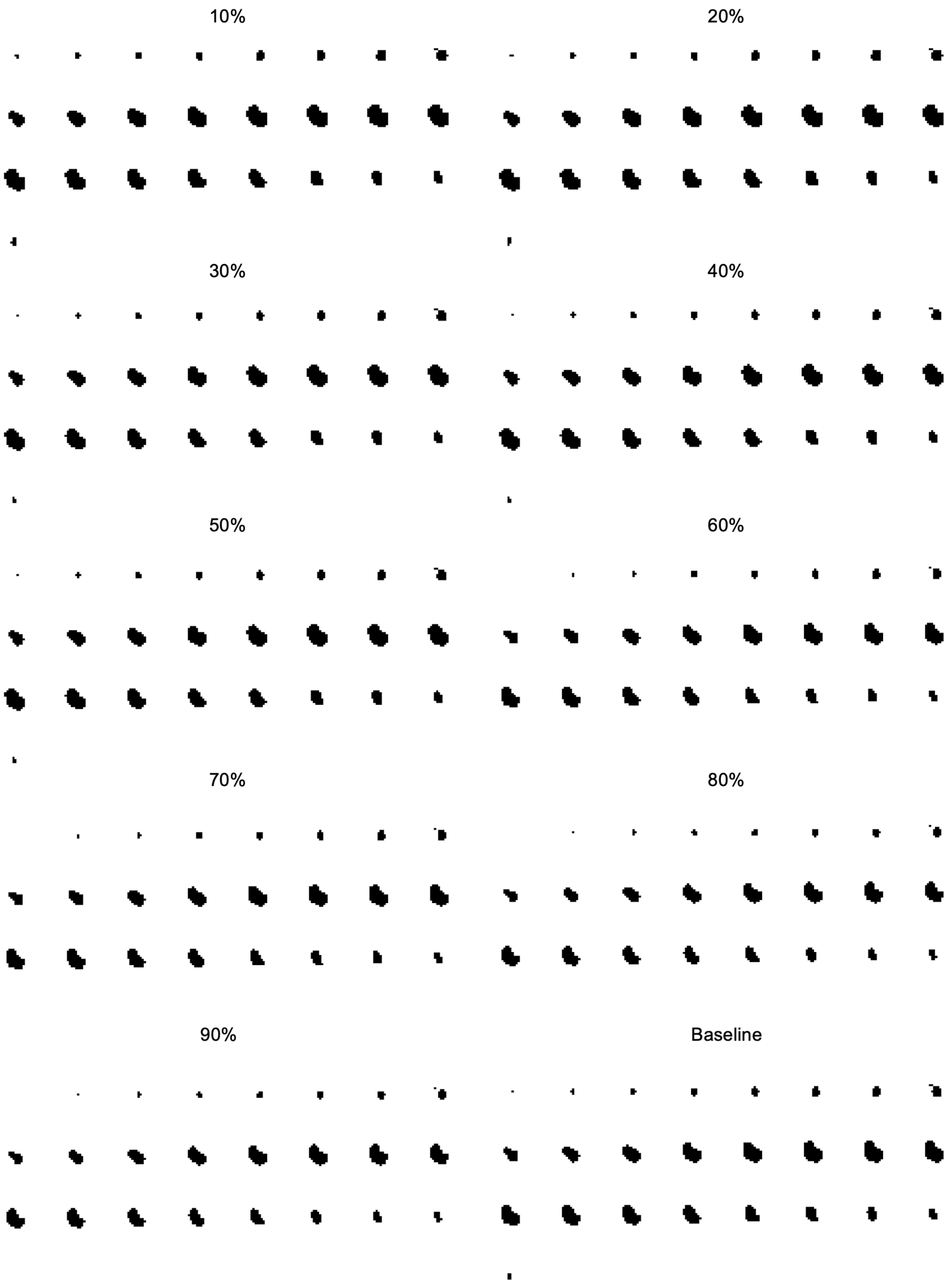

Figure 16: The effect of the super-sampling threshold on the mask boundary is shown for every single PET ROI for STS case 15. The baseline ROI is also reported.

F Hierarchical clustering

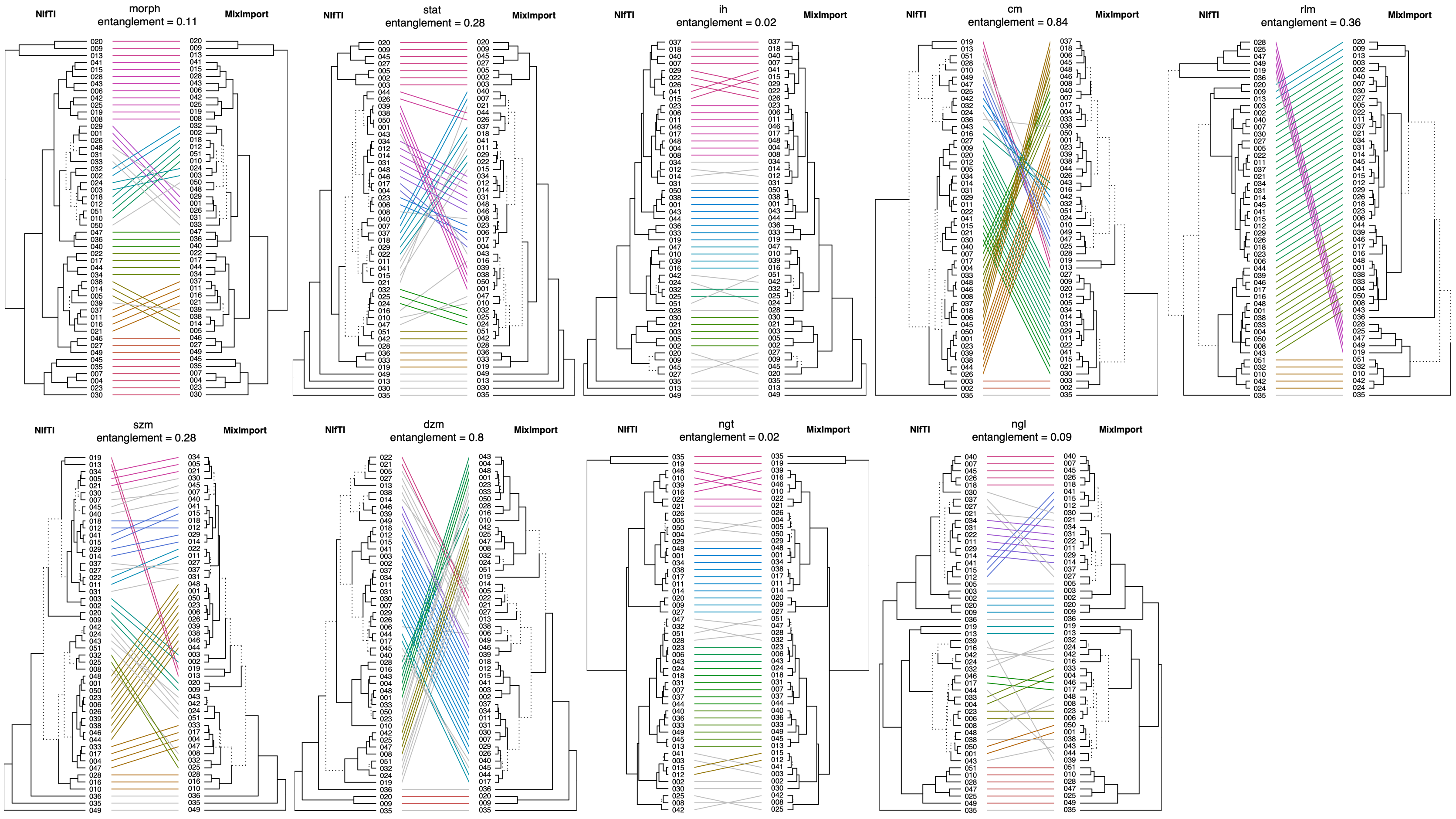

Figure 17: Comparison of hierarchical clustering of patients for different families of features, using the mixed dataset (combining different import strategies) compared to the baseline. The 9 feature families: Morphology (morph), Intensity based Statistics (stat), Intensity Histogram (ih), Gray Level Co-occurrence Matrix (cm), Gray Level Run Length Matrix (rlm), Gray Level Size Zone Matrix (szm), Gray Level Distance Zone Matrix (dzm), Neighbouring Gray Tone Difference Matrix (ngt), and Neighbourhood Gray Level Dependence Matrix (ngl). Hierarchical clustering using the baseline features (left dendrogram) was compared to the mixed results (right dendrogram), for CT.

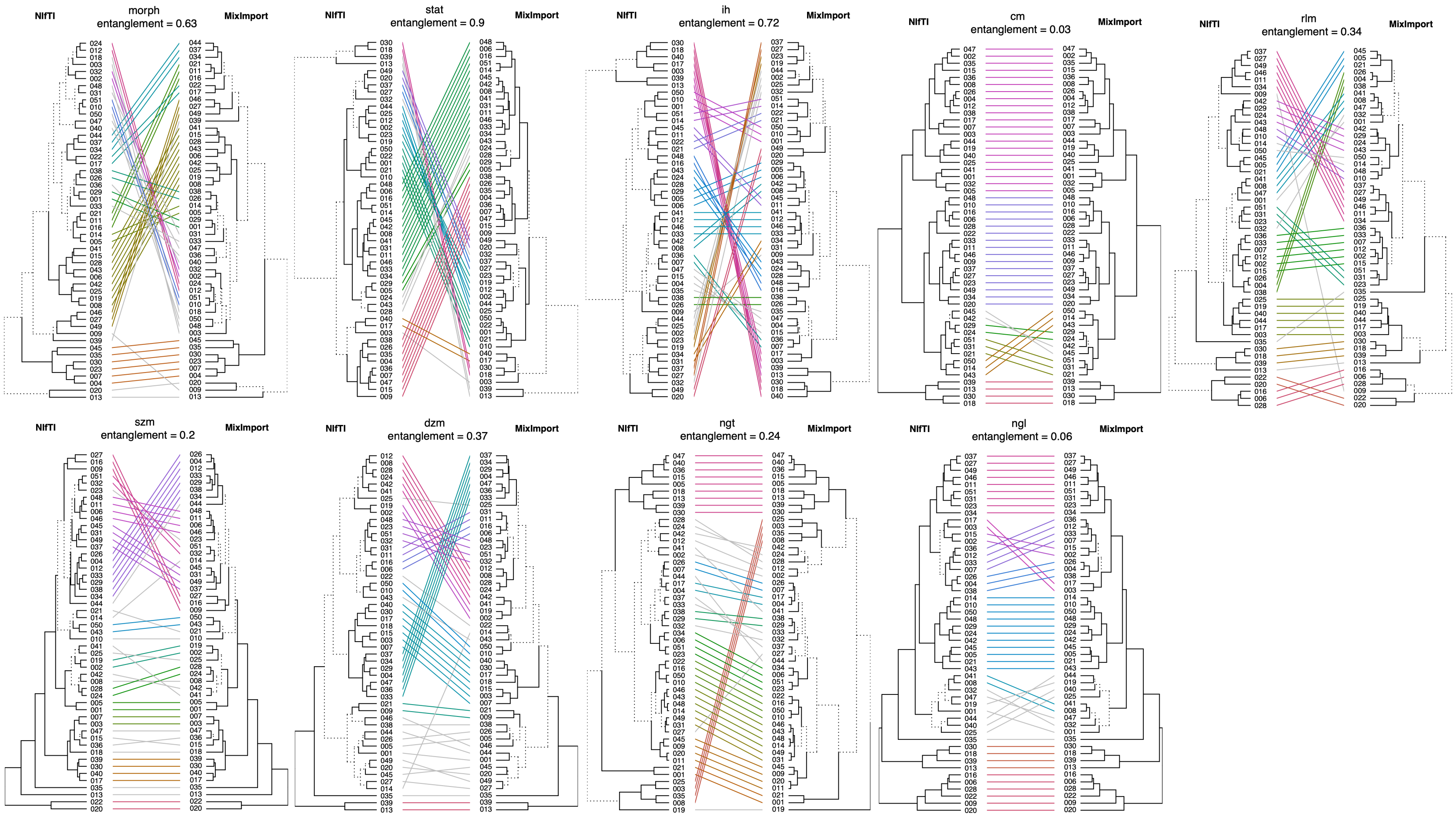

Figure 18: Comparison of hierarchical clustering of patients for different families of features, using the mixed dataset (combining different import strategies) compared to the baseline. The 9 feature families: Morphology (morph), Intensity based Statistics (stat), Intensity Histogram (ih), Gray Level Co-occurrence Matrix (cm), Gray Level Run Length Matrix (rlm), Gray Level Size Zone Matrix (szm), Gray Level Distance Zone Matrix (dzm), Neighbouring Gray Tone Difference Matrix (ngt), and Neighbourhood Gray Level Dependence Matrix (ngl). Hierarchical clustering using the baseline features (left dendrogram) was compared to the mixed results (right dendrogram), for MRI.

G Extraction details

Based on the IBSI reporting checklist documentation. Only relevant items for this study are included below. For extraction details also see section A.

topic	details
Region of interest	Soft-tissue sarcoma (STS)
Patient imaging	This study uses a publicly available dataset. See [1] for relevant acquisition infomation for each of the 3 imaging modalities. We used the dataset as it was prepared for the IBSI standardisation validation phase [2], which preprocessed the images.
Image interpolation	No interpolation was used as the study aimed to examine only the effect of each software's mask conversion.
Resegmentation thresholds	CT: [-1000, inf], MRI [0, inf], PET: [0, inf]
Discretisation method	Fixed Bin Size (FBS)<br>CT: FBS: 10<br>MRI: FBS: 10<br>PET: FBS: 0.25
IBSI compliance	Software matched all benchmarks.
Software availability	Currently in-house.
**Texture parameters**	
Texture matrix aggregation	merged
Distance weighting	no weighting.
CM symmetry	symmetric matrices used
CM distance	1
SZM linkage distance	Chebyshev distance of 1
DZM linkage distance	Chebyshev distance of 1
DZM zone distance norm	Chebyshev distance of 1
NGTDM distance	Chebyshev distance of 1
NGLDM distance	Chebyshev distance of 1
NGLDM coarseness	0

Table 1: Processing details.

Feature tag	Feature name (IBSI)
**Morphology**	
morph_ volume	Morphology Volume (mesh)
morph_ vol_ approx	Volume (voxel counting)
morph_ area_ mesh	Surface area (mesh)
morph_ av	Surface to volume ratio
morph_ comp_ 1	Compactness 1
morph_ comp_ 2	Compactness 2
morph_ sph_ dispr	Spherical disproportion
morph_ sphericity	Sphericity
morph_ asphericity	Asphericity

morph_ com	Centre of mass shift
morph_ diam	Maximum 3D diameter
morph_ pca_ maj_ axis	Major axis length
morph_ pca_ min_ axis	Minor axis length
morph_ pca_ least_ axis	Least axis length
morph_ pca_ elongation	Elongation
morph_ pca_ flatness	Flatness
morph_ vol_ dens_ aabb	Volume density (AABB)
morph_ area_ dens_ aabb	Area density (AABB)
morph_ vol_ dens_ aee	Volume density (AEE)
morph_ area_ dens_ aee	Area density (AEE)
morph_ vol_ dens_ conv_ hull	Volume density (convex hull)
morph_ area_ dens_ conv_ hull	Area density (convex hull)
morph_ integ_ int	Integrated intensity
**Statistics**	
stat_ mean	Mean
stat_ var	Variance
stat_ skew	Skewness
stat_ kurt	(Excess) kurtosis
stat_ median	Median
stat_ min	Minimum
stat_ p10	10th percentile
stat_ p90	90th percentile
stat_ max	Maximum
stat_ iqr	Interquartile range
stat_ range	Range
stat_ mad	Mean absolute deviation
stat_ rmad	Robust mean absolute deviation
stat_ medad	Median absolute deviation
stat_ cov	Coefficient of variation
stat_ qcod	Quartile coefficient of dispersion
stat_ energy	Energy
stat_ rms	Root mean square
**Intensity histogram**	
ih_ mean	Intensity histogram Mean
ih_ var	Variance
ih_ skew	Skewness
ih_ kurt	(Excess) kurtosis
ih_ median	Median
ih_ min	Minimum
ih_ p10	10th percentile
ih_ p90	90th percentile
ih_ max	Maximum
ih_ mode	Mode
ih_ iqr	Interquartile range
ih_ range	Range

ih_mad	Mean absolute deviation
ih_rmad	Robust mean absolute deviation
ih_medad	Median absolute deviation
ih_cov	Coefficient of variation
ih_qcod	Quartile coefficient of dispersion
ih_entropy	Entropy
ih_uniformity	Uniformity
ih_max_grad	Maximum histogram gradient
ih_max_grad_g	Maximum histogram gradient intensity
ih_min_grad	Minimum histogram gradient
ih_min_grad_g	Minimum histogram gradient intensity
**Co-occurrence matrix (3D merged)**	
cm_joint_max_3D_comb	Joint maximum
cm_joint_avg_3D_comb	Joint average
cm_joint_var_3D_comb	Joint variance
cm_joint_entr_3D_comb	Joint entropy
cm_diff_avg_3D_comb	Difference average
cm_diff_var_3D_comb	Difference variance
cm_diff_entr_3D_comb	Difference entropy
cm_sum_avg_3D_comb	Sum average
cm_sum_var_3D_comb	Sum variance
cm_sum_entr_3D_comb	Sum entropy
cm_energy_3D_comb	Angular second moment
cm_contrast_3D_comb	Contrast
cm_dissimilarity_3D_comb	Dissimilarity
cm_inv_diff_3D_comb	Inverse difference
cm_inv_diff_norm_3D_comb	Normalised inverse difference
cm_inv_diff_mom_3D_comb	Inverse difference moment
cm_inv_diff_mom_norm_3D_comb	Normalised inverse difference moment
cm_inv_var_3D_comb	Inverse variance
cm_corr_3D_comb	Correlation
cm_auto_corr_3D_comb	Autocorrelation
cm_clust_tend_3D_comb	Cluster tendency
cm_clust_shade_3D_comb	Cluster shade
cm_clust_prom_3D_comb	Cluster prominence
cm_info_corr1_3D_comb	Information correlation 1
cm_info_corr2_3D_comb	Information correlation 2
**Run length matrix (3D merged)**	
rlm_sre_3D_comb	Short runs emphasis
rlm_lre_3D_comb	Long runs emphasis
rlm_lgre_3D_comb	Low grey level run emphasis
rlm_hgre_3D_comb	High grey level run emphasis
rlm_srlge_3D_comb	Short run low grey level emphasis
rlm_srhge_3D_comb	Short run high grey level emphasis
rlm_lrlge_3D_comb	Long run low grey level emphasis
rlm_lrhge_3D_comb	Long run high grey level emphasis

rlm_ glnu_ 3D_ comb	Grey level non-uniformity
rlm_ glnu_ norm_ 3D_ comb	Normalised grey level non-uniformity
rlm_ rlnu_ 3D_ comb	Run length non-uniformity
rlm_ rlnu_ norm_ 3D_ comb	Normalised run length non-uniformity
rlm_ r_ perc_ 3D_ comb	Run percentage
rlm_ gl_ var_ 3D_ comb	Grey level variance
rlm_ rl_ var_ 3D_ comb	Run length variance
rlm_ rl_ entr_ 3D_ comb	Run entropy
**Size zone matrix (3D)**	
szm_ sze_ 3D	Small zone emphasis
szm_ lze_ 3D	Large zone emphasis
szm_ lgze_ 3D	Low grey level emphasis
szm_ hgze_ 3D	High grey level emphasis
szm_ szlge_ 3D	Small zone low grey level emphasis
szm_ szhge_ 3D	Small zone high grey level emphasis
szm_ lzlge_ 3D	Large zone low grey level emphasis
szm_ lzhge_ 3D	Large zone high grey level emphasis
szm_ glnu_ 3D	Grey level non-uniformity
szm_ glnu_ norm_ 3D	Normalised grey level non-uniformity
szm_ zsnu_ 3D	Zone size non-uniformity
szm_ zsnu_ norm_ 3D	Normalised zone size non-uniformity
szm_ z_ perc_ 3D	Zone percentage
szm_ gl_ var_ 3D	Grey level variance
szm_ zs_ var_ 3D	Zone size variance
szm_ zs_ entr_ 3D	Zone size entropy
**Distance zone matrix**	
dzm_ sde_ 3D	(3D) Small distance emphasis
dzm_ lde_ 3D	Large distance emphasis
dzm_ lgze_ 3D	Low grey level emphasis
dzm_ hgze_ 3D	High grey level emphasis
dzm_ sdlge_ 3D	Small distance low grey level emphasis
dzm_ sdhge_ 3D	Small distance high grey level emphasis
dzm_ ldlge_ 3D	Large distance low grey level emphasis
dzm_ ldhge_ 3D	Large distance high grey level emphasis
dzm_ glnu_ 3D	Grey level non-uniformity
dzm_ glnu_ norm_ 3D	Normalised grey level non-uniformity
dzm_ zdnu_ 3D	Zone distance non-uniformity
dzm_ zdnu_ norm_ 3D	Normalised zone distance non-uniformity
dzm_ z_ perc_ 3D	Zone percentage
dzm_ gl_ var_ 3D	Grey level variance
dzm_ zd_ var_ 3D	Zone distance variance
dzm_ zd_ entr_ 3D	Zone distance entropy
**Neighbourhood grey tone difference matrix (3D)**	
ngt_ coarseness_ 3D	Coarseness
ngt_ contrast_ 3D	Contrast
ngt_ busyness_ 3D	Busyness

ngt_ complexity_ 3D	Complexity
ngt_ strength_ 3D	Strength
**Neighbouring grey level dependence matrix (3D)**	
ngl_ lde_ 3D	Low dependence emphasis
ngl_ hde_ 3D	High dependence emphasis
ngl_ lgce_ 3D	Low grey level count emphasis
ngl_ hgce_ 3D	High grey level count emphasis
ngl_ ldlge_ 3D	Low dependence low grey level emphasis
ngl_ ldhge_ 3D	Low dependence high grey level emphasis
ngl_ hdlge_ 3D	High dependence low grey level emphasis
ngl_ hdhge_ 3D	High dependence high grey level emphasis
ngl_ glnu_ 3D	Grey level non-uniformity
ngl_ glnu_ norm_ 3D	Normalised grey level non-uniformity
ngl_ dcnu_ 3D	Dependence count non-uniformity
ngl_ dcnu_ norm_ 3D	Normalised dependence count non-uniformity
ngl_ gl_ var_ 3D	Grey level variance
ngl_ dc_ var_ 3D	Dependence count variance
ngl_ dc_ entr_ 3D	Dependence count entropy
ngl_ dc_ energy_ 3D	Dependence count energy

Table 2: Feature tags and names.

References

[1] M. Vallières, C. R. Freeman, S. R. Skamene, and I. El Naqa, "A radiomics model from joint FDG-PET and MRI texture features for the prediction of lung metastases in soft-tissue sarcomas of the extremities," 2015. Version Number: 1 Type: dataset.

[2] A. Zwanenburg, M. Vallières, M. A. Abdalah, H. J. W. L. Aerts, V. Andrearczyk, A. Apte, S. Ashrafinia, S. Bakas, R. J. Beukinga, R. Boellaard, M. Bogowicz, L. Boldrini, I. Buvat, G. J. R. Cook, C. Davatzikos, A. Depeursinge, M.-C. Desseroit, N. Dinapoli, C. V. Dinh, S. Echegaray, I. El Naqa, A. Y. Fedorov, R. Gatta, R. J. Gillies, V. Goh, M. Götz, M. Guckenberger, S. M. Ha, M. Hatt, F. Isensee, P. Lambin, S. Leger, R. T. Leijenaar, J. Lenkowicz, F. Lippert, A. Losnegård, K. H. Maier-Hein, O. Morin, H. Müller, S. Napel, C. Nioche, F. Orlhac, S. Pati, E. A. Pfaehler, A. Rahmim, A. U. Rao, J. Scherer, M. M. Siddique, N. M. Sijtsema, J. Socarras Fernandez, E. Spezi, R. J. Steenbakkers, S. Tanadini-Lang, D. Thorwarth, E. G. Troost, T. Upadhaya, V. Valentini, L. V. van Dijk, J. van Griethuysen, F. H. van Velden, P. Whybra, C. Richter, and S. Löck, "The Image Biomarker Standardization Initiative: Standardized Quantitative Radiomics for High-Throughput Image-based Phenotyping," *Radiology*, vol. 295, pp. 328–338, May 2020.
